# Enhancing Photostability by Thermodynamic and Kinetic Factors: Free-Base and Palladium *meso*-Aryl-octaethylporphyrins

**DOI:** 10.1021/acs.jpcb.5c02423

**Published:** 2025-05-28

**Authors:** Maciej Banaszek, Barbara Golec, Renata Rybakiewicz-Sekita, Jarosław Kowalski, Piotr Szczodry, Natalia Dutkiewicz, Jacek Waluk, Aleksander Gorski

**Affiliations:** † Faculty of Mathematics and Science, Cardinal Stefan Wyszyński University, Dewajtis 5, Warsaw 01-815, Poland; ‡ Institute of Physical Chemistry, Polish Academy of Sciences, Kasprzaka 44/52, Warsaw 01-224, Poland

## Abstract

Photostability is a crucial parameter in applications based on light–matter interactions. In this work, we demonstrate that photodegradation efficiency can be strongly decreased by altering the thermodynamic and kinetic characteristics of a chromophore. Photobleaching quantum yields have been determined for a series of free-base octaethylporphyrins and their palladium metallocomplexes gradually substituted by phenyl groups at the *meso* positions. Due to increased oxidation potential of palladium porphyrins, photostability is improved in comparison with zinc or magnesium derivatives. A spectacular effect is observed for nonplanar palladium derivatives in which the triplet lifetime in deoxygenated solution is shortened by 3 orders of magnitude with respect to planar porphyrins. Comparison of photodestruction efficiencies in oxygen-containing and degassed toluene samples shows a hundred-fold decrease of photobleaching quantum yields for nonplanar palladium porphyrins, reaching an extremely low value of less than 10^–9^. In contrast, free-base, nonplanar porphyrins are less stable than the planar analogues in nondegassed toluene. Finally, planar free-base and palladium porphyrins become significantly less photostable in the degassed solution because the triplet decay time increases by 3 orders of magnitude compared to oxygen-containing samples.

## Graphical abstract

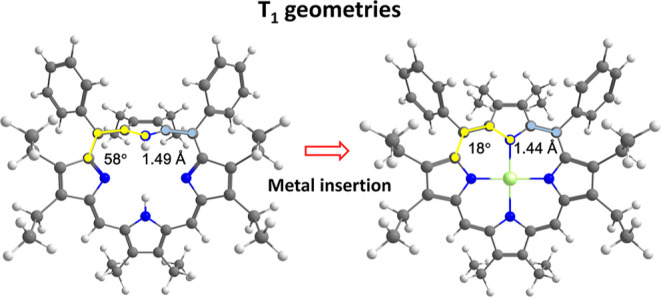

## 1 Introduction

Studies of the photochemistry and supramolecular chemistry of porphyrin derivatives have been actively developed over the past few decades. Porphyrins are well-known for their diverse functionality in various natural processes, including respiration,^1^ electron transfer,^2^ oxidation catalysis,^3^ and photosynthesis.^4^ The ability of porphyrins to generate singlet oxygen finds applications in medicine for photodynamic therapy (PDT)^5^ and in biology for the photoinactivation of bacteria.^6^ The crucial parameters for a variety of applications that use porphyrins as photosensitizers are the quantum yield of singlet oxygen formation and the photostability. In addition to the fundamental parameters of the porphyrin macrocycle, substitution of peripheral groups and coordination of heavy atoms serve as tools for fine-tuning the excited-state properties of porphyrins, influencing their photostability,^7^ and increasing their ability of generating singlet oxygen with close to 100% efficiency. Therefore, it is essential to gain insight into the specific factors that may control the photophysical properties of individual porphyrin subunits.^8^

In this study, we investigate the photodegradation of a series of free-base and Pd-octaethylporphyrin (PdOEP) derivatives functionalized with an increasing number (referred to as “*n*” in this article) of bulky *meso*-phenyl substituents (Figure 1). Additionally, octaethylporphyrin (OEP), tetraphenylporphyrin (TPP), and their palladium metallocomplexes are considered as reference compounds. The objectives of this research are 2-fold: (i) to identify the products of photodegradation; (ii) to assess the role of thermodynamic and kinetic factors in the photostability in oxygen and oxygen-free atmospheres by analyzing photophysical parameters such as triplet state lifetimes and quantum yields of singlet oxygen generation.

**Figure 1:**
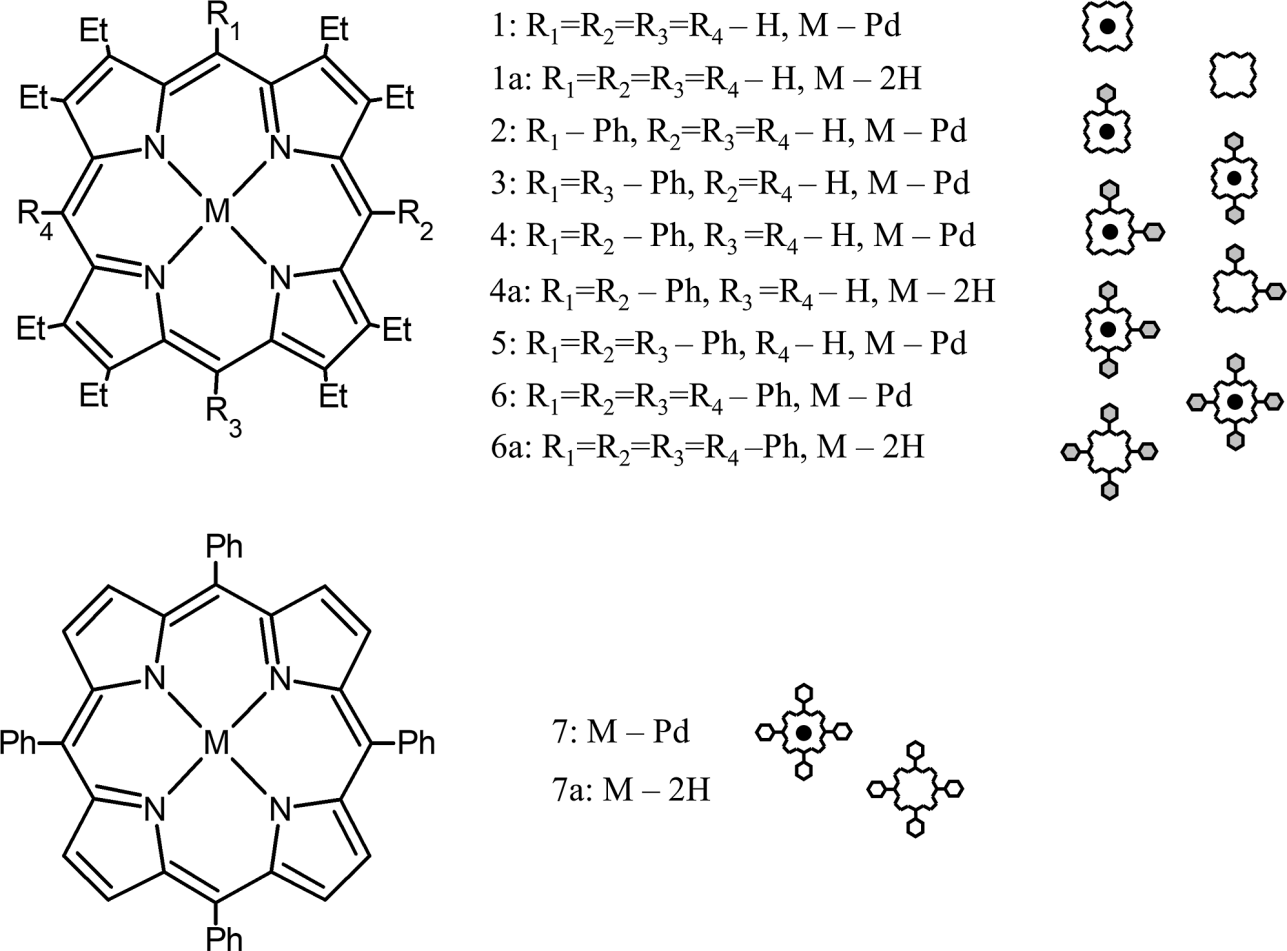
Top: chemical structures and pictograms for a series of *meso*-phenyl-substituted derivatives of octaethylporphyrin. Bottom: chemical structures and pictograms for derivatives of tetraphenylporphyrin. **1**: palladium octaethylporphyrin (PdOEP); **1a**: octaethylporphyrin (OEP); **2**: palladium 5-phenyl-2,3,7,8,12,13,17,18-octaethylporphyrin (PdOEP1); **3**: palladium 5,15-diphenyl-2,3,7,8, 12,13,17,18-octaethylporphyrin (PdOEP2t); **4**: palladium 5,10-diphenyl-2,3,7,8,12,13,17,18-octaethylporphyrin (PdOEP2c); **4a**: 5,10-diphenyl-2,3,7,8,12,13,17,18-octaethylporphyrin (OEP2c); **5**: palladium 5,10,15-triphenyl-2,3,7,8,12,13,17,18-octaethylporphyrin (PdOEP3); **6**: palladium 5,10,15,20-tetraphenyl-2,3,7,8,12,13,17,18-octaethylporphyrin (PdOEP4); **6a**: 5,10,15,20-tetraphenyl-2,3,7,8,12,13,17,18-octaethylporphyrin (OEP4); **7**: palladium tetraphenylporphyrin (PdTPP); **7a**: tetraphenylporphyrin (TPP).

## 2 Experimental Section

### 2.1 Reagents

The investigated porphyrins were obtained as described previously.^8,9^ Toluene (spectroscopic grade) and phenalenone (97% grade) purchased from Merck were used without further purification.

### 2.2 Deaerated Samples

The samples were deaerated before measurements by a freeze–pump–thaw method. At least seven freeze–pump–thaw cycles were performed before each lifetime experiment. The last pumping cycle was performed at the pressure of 4 × 10^–5^ mbar.

### 2.3 Electronic Absorption Spectra

The UV–vis spectra were measured using a Shimadzu UV 2700 spectrophotometer.

### 2.4 Triplet State Lifetimes

The triplet state lifetimes were determined using a home-built setup for transient absorption measurement in nano- to milliseconds time ranges. An Opotek Radiant 355 laser (210–2500 nm tuning spectral region, 5 ns pulse width, 10 Hz repetition rate) was used as the excitation source. All compounds were excited in the region of the Soret band (385–430 nm, and the pulse energy was in the range of 30–200 μJ). The continuous output of a laser-driven Xe lamp (Energetiq EQ-99-Plus-EU) was used as the probe light source. The setup was equipped with a Hamamatsu R955 photomultiplier and a Yokogawa DL9140 fast oscilloscope. A special 1 cm sealed quartz cuvette with a high-vacuum valve (max pressure: 10^–7^ mbar) was used for deaerated samples. The experiments were conducted at a low concentration of the investigated compounds, close to 1 × 10^–6^ M, to avoid triplet–triplet annihilation processes.

### 2.5 Singlet Oxygen Quantum Yield

The quantum yield of singlet oxygen generation, Φ_Δ_, was determined with a home-built setup. An Opotek Radiant 355 laser was used for excitation at 370 nm. The emission was detected using a BENTHAM DTMc300 double monochromator, equipped with a Hamamatsu H10330C-75 thermoelectrically cooled photomultiplier. To determine Φ_Δ_, amplitudes of singlet oxygen phosphorescence decay curves were measured at the emission maximum (1275 nm). Singlet oxygen was generated by the standard (signal amplitude *A*_0_) and by the compound under investigation (amplitude *A*_*x*_). Both samples were compared using the same solvent (toluene). Φ_Δ_ = Φ^0^_Δ_×*A*_*x*_×(1–10^–OD^_0_)/(*A*_0_×(1–10^–OD^_*x*_)), where 1–10^–OD^_0_ and 1–10^–OD^_*x*_ correspond to fractions of light absorbed by the phenalenone and the measured porphyrin derivative, respectively, at a given excitation wavelength. At room temperature, the quantum yield of singlet oxygen generation for phenalenone in toluene is close to 1.^10^ We used the value of 1.0 in the analyses.

### 2.6 Photodegradation Quantum Yield

The porphyrins dissolved in toluene were irradiated in quartz 1 cm cuvettes using two different Thorlabs high-power LEDs: M385L2 (measured wavelength maximum at 388 nm, 102 mW) and M420L2 (measured maximum at 419 nm, 170 mW). The photodegradation quantum yield was determined by measuring the sample absorbance before irradiation (*A*_0_) and at time *t* after the beginning of irradiation (*A*(*t*)). Next, the *A*_0_/*A*(*t*) ratio was plotted as a function of *F*(*t*), defined as *N*_tot_(*t*)/*A*(*t*), where *N*_tot_(*t*) denotes the total number of photons absorbed after irradiating the sample for time *t.* The photobleaching quantum yield (Φ_pb_) was calculated based on the following equation: Φ_pb_ = (*b* × *N*_Av_ × *V*)/(1000 × ε × l), where *b* is the slope in the equation: *A*_0_/*A*(*t*) = 1+b*F*(*t*), *N*_Av_ is the Avogadro number, *V* is the sample volume (in mL), ε is the molar absorption coefficient at the wavelength selected to monitor absorbance decrease, and *l* is the optical path length (in cm). The detailed procedure for the determination and calculation of the quantum yield of photodegradation has been described elsewhere.^11^

### 2.7 Mass Spectrometry Analyses

Mass spectra were obtained using a Synapt G2-S mass spectrometer (Waters) equipped with the atmospheric pressure chemical ionization ion source (APCI) and quadrupole-time-of-flight (TOF) mass analyzer. The resolving power of the TOF analyzer was 30,000 fwhm. Methanol was used as a mobile phase with a 100 μL/min flow rate. The measurement was performed both in the positive and negative ion modes. The measurement in positive mode was performed with corona current set to 13.0 μA. The desolvation gas flow was 600 L/h and the probe temperature was 550 °C. The sampling cone voltage and source offset were set to 40 V and the source temperature was 120 °C. The measurement in negative ion mode was performed with corona current set to 12.0 μA. The desolvation gas flow was 600 L/h and the probe temperature was 550 °C. The sampling cone voltage and source offset were set to 20 V and the source temperature was 120 °C. The Leucine-Enkephalin solution was used as the Lock-Spray reference material. The instrument was controlled and the recorded data were processed using the MassLynx V4.2 software package (Waters).

### 2.8 Quantum-Chemical Calculations

Calculations were performed with Gaussian 16 (C.01) quantum-chemical software packages.^12^ The Becke–Lee–Yang–Parr exchange–correlation three-parameter functional (B3LYP) and def2-SVP basis set were used.

### 2.9 Electrochemistry

Cyclic voltammograms were recorded using a three-electrode electrochemical cell equipped with a platinum disc working electrode (1.6 mm diameter), a platinum wire counter electrode, and a Ag/AgCl reference electrode. The studied compound was dissolved in a 0.1 M Bu_4_NPF_6_/THF electrolyte to yield a saturated solution. All cyclic voltammetry (CV) measurements were performed with a VSP electrochemistry system from BioLogic Science Instruments, controlled by EC-Lab software from the same manufacturer.

## 3 Results

### 3.1 Photostability

The changes in electronic absorption spectra during irradiation with UV light of nondegassed samples of all studied porphyrins dissolved in toluene suggest their decomposition (Figures 2 and S1). In all cases, the absorption of the substrate decreases with the duration of irradiation. A closer examination of the differential absorption spectra obtained after subtracting the absorption spectrum of substrate allows for identifying band patterns corresponding to photoproducts. The differential absorption spectrum of PdOEP4 (Figure 2) exhibits two positive broad bands attributed to the photoproduct. The first band appears above 800 nm, a region where no substrate absorption occurs, making the band clearly visible. This new band, with a maximum at 850 nm, is distinctly observed in the full electronic absorption spectrum of the PdOEP4 photoproduct, presented in the Supporting Information, Figure S2. The second band is located below 650 nm.

**Figure 2:**
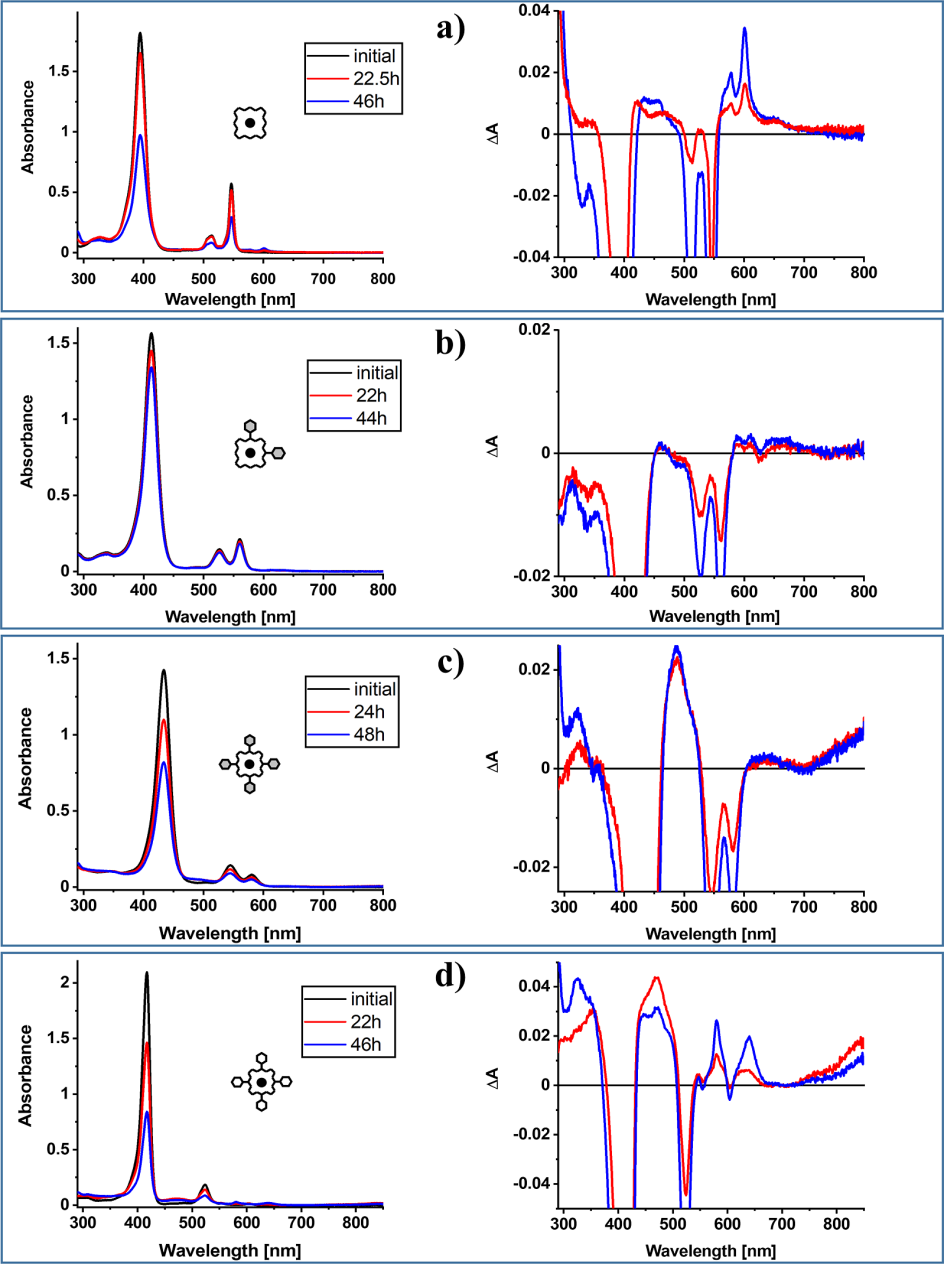
Absorption spectra in nondegassed toluene; from top to bottom: PdOEP (a), PdOEP2c (b), PdOEP4 (c), and PdTTPP (d). Samples were irradiated with two LEDs (388 nm maximum, power of 102 mW for PdOEP, and 419 nm maximum, power of 170 mW for the rest of compounds). Left, changes in the absorption spectra. Right, differential absorption spectra obtained after subtracting the absorption spectrum of substrate.

The differential absorption spectrum of PdOEP3 (Figures S1 and S3), PdOEP2t (Figure S1), PdOEP1 (Figure S1), and PdTPP (Figure 2) resembles that of PdOEP4. In the case of the high-energy absorption bands assigned to photoproducts, their overlap with the absorption bands of the initial compounds complicates the overall picture. However, the photoproduct bands remain distinguishable. The differential absorption spectra of PdOEP (Figure 2) and PdOEP1 (Figure S1) differ from those of the other compounds, as no new bands were observed at around 800 nm. Extended irradiation (resulting in 47% and 60% decomposition for PdOEP and PdOEP1, respectively) leads to the appearance of new bands below 300 nm. The changes in the absorption spectrum of PdOEP2c (Figure 2) occur the slowest in the entire series, with only 15% conversion observed after 44 h of irradiation. The differential absorption spectrum of PdOEP2c is not particularly distinct, as the band around 800 nm is very weak. However, the overall spectrum more closely resembles that of PdOEP4 rather than that of PdOEP.

In the degassed toluene sample of PdOEP, new absorption bands appear at <300, 412, 600, and 614 nm (Figure S4). For PdTPP, new bands are observed at 340, 434, 600, and 826 nm. The band at 826 nm is weaker than those in the rest of the spectrum. Among the phenyl-substituted derivatives of PdOEP, only PdOEP2t shows clearly visible changes in the electronic absorption spectra under irradiation. New bands appear at <300, 431, and 600 nm (Figure S4). The changes in absorption spectra for the rest of compounds in degassed toluene are minor due to increased photostability and insignificant spectral changes even after prolonged irradiation.

Based on the changes in absorption during irradiation and the known power of the light source, we established the values of photodegradation quantum yields. The obtained values of Φ_pb_ are listed in Table 1. The highest photostability was noted for PdOEP2c (Φ_pb_∼0.8 × 10^–7^) and two lowest values for PdOEP1 and PdOEP2t (Φ_pb_∼6.4 × 10^–7^).

**Table 1:**
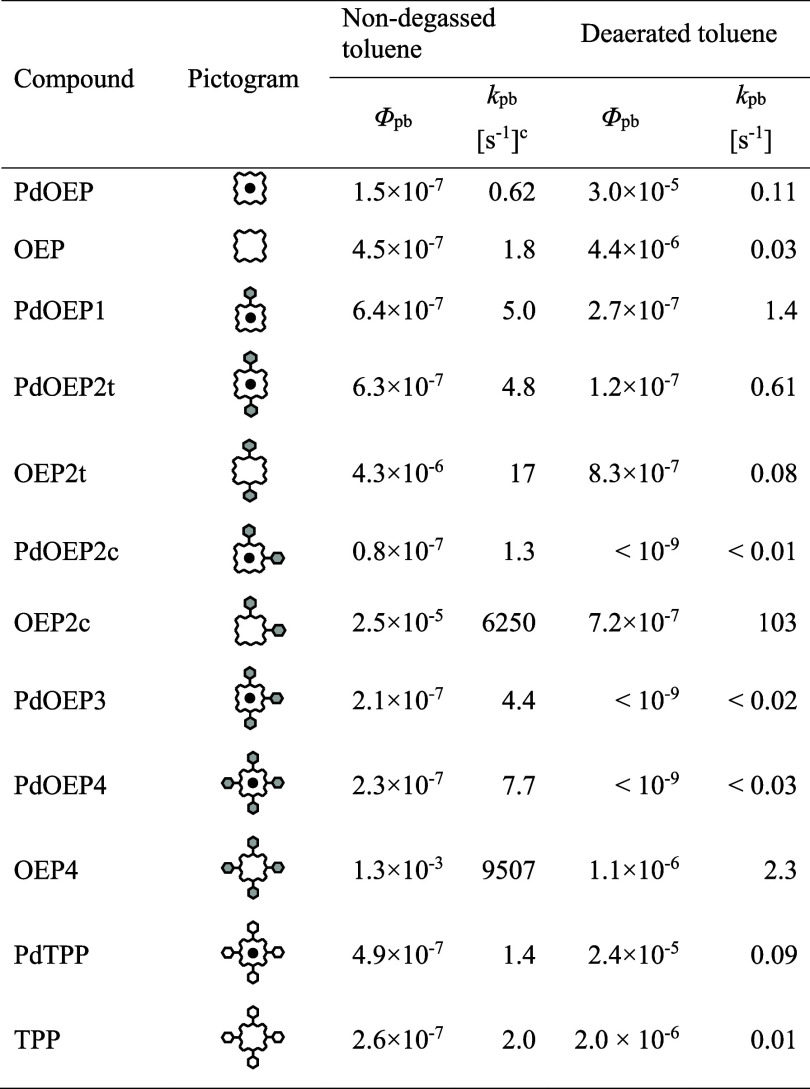
Quantum Yields of Photodegradation, Φ_pb_ ^a^, and Photobleaching Rates, *k*_pb_ ^b^, Determined for a Series of OEP and PdOEP Derivatives, and PdTPP Obtained in Nondegassed and Deaerated Toluene

a Estimated error: ±30%.

b *k*_pb_ = Φ_pb_/(Φ_T_×τ_T_); τ_T_ is the triplet lifetime and Φ_T_ is the triplet formation yield.

c The triplet formation yield was assumed to be close to unity for all palladium metallocomplexes, and for metal-free compounds, Φ_T_ was assumed to be equal to Φ_Δ_.

In deaerated toluene, the quantum yields of decomposition decrease significantly for derivatives with two or more phenyls substituted at the *meso* position. PdOEP2c, PdOEP3, and PdOEP4 become more than 2 orders of magnitude photostable than in a nondegassed solution (Table 1). Contrary to the stability of phenyl-substituted PdOEP derivatives, the photostability of the two reference compounds in degassed solution decreases by 50 and 200 times for PdTPP and PdOEP, respectively.

To highlight the influence of the inner heavy ion (Pd) on the photophysics and photostability of the investigated series of compounds, additional experiments were carried out on free-base porphyrins: OEP, OEP2t, OEP2c, OEP4, and TPP (Figure 1). The spectral changes in absorption observed for free-base porphyrins upon UV irradiation are presented in the Supporting Information (Figure S5).

The photostability of the latter in nondegassed toluene gradually decreases in the following order: TPP, OEP, OEP2t, OEP2c, and OEP4 (Table 1), starting from 2.6 × 10^–7^ for TPP and reaching the lowest value in the series of 1.3 × 10^–3^ for OEP4. In deaerated toluene, the photostability of OEP and TPP decreases by nearly 1 order of magnitude compared to that in nondegassed toluene. In contrast, in deaerated toluene, the remaining free-base porphyrins exhibit significant improvement in photostability: approximately 5-fold for OEP2t, 35-fold for OEP2c, and more than 1000-fold for OEP4.

### 3.2 Triplet Lifetimes and Singlet Oxygen Generation

The triplet lifetimes and the values of singlet oxygen formation yields obtained for nondegassed and deaerated toluene are listed in Table 2.

**Table 2:**
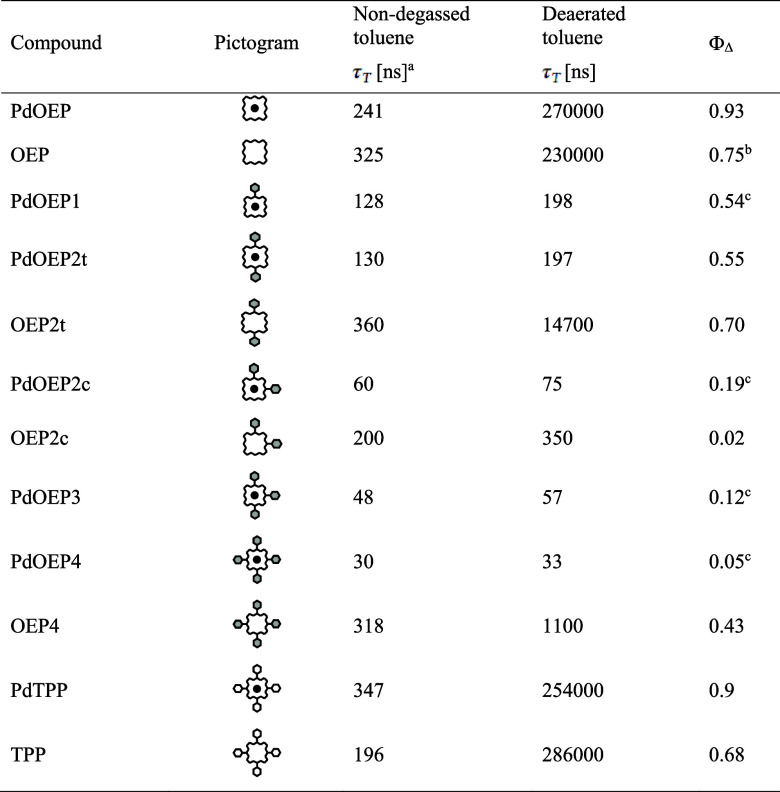
Triplet Lifetimes and Quantum Yield of Singlet Oxygen Generation of a Series of PdOEP Derivatives and PdTPP Obtained in Nondegassed and Deaerated Toluene

a Estimated error: ±5%.

b Ref 23.

c Ref 8.

The triplet lifetimes in nondegassed solutions span the range of 347 to 30 ns. The highest values are observed for parent PdTPP (347 ns) and PdOEP (241 ns). For the phenyl-substituted PdOEPs, the triplet lifetime decreases to about 130 ns for PdOEP1 and PdOEP2t and then to 60, 48, and 30 ns for PdOEP2c, PdOEP3, and PdOEP4, respectively. The difference in lifetimes between PdOEP2t and PdOEP2c suggests that the decrease of the triplet lifetime is connected not only with the increasing number of phenyl substituents, but also with the geometry of the compound.

The observed triplet lifetime values correlate well with the singlet oxygen generation yields. In PdTPP and PdOEP, Φ_Δ_ is as high as 0.9 and 0.93, respectively. For PdOEP1 and PdOEP2t, these values are reduced to 0.54 and 0.55. For PdOEP2c, PdOEP3, and PdOEP4, they decreased to 0.19, 0.15, and 0.05, respectively.

In the deaerated solvent, the differences between the τ_*T*_ values of the reference compounds and the phenyl-substituted PdOEP derivatives are enormous. For PdTPP and PdOEP, the triplet lifetimes are as high as 254,000 and 270,000 ns, respectively, whereas for the phenyl-substituted PdOEP porphyrins, they are comparable with the lifetimes observed in nondegassed toluene (τ_*T*_ from 33 to 198 ns).

The triplet states of free-base OEP derivatives and TPP in nondegassed toluene live for several hundred nanoseconds. In deaerated toluene, the triplet lifetimes of OEP2t, OEP2c, and OEP4 increase to 14700, 350, and 1100 ns, respectively (Table 2). It is remarkable that the lengthening of the triplet lifetime is much larger for the OEP2t compared to the other two compounds. The triplet lifetimes of the OEP and TPP in deaerated toluene increase to several hundred microseconds, similar to the case of their palladium metallocomplexes.

### 3.3 Photoproducts

To evaluate the nature of the photoproducts in nondegassed toluene, the mass spectra of compounds obtained after irradiation were compared with those of the substrates (Figures S6–S19). For all investigated complexes with phenyl groups substituted at the *meso* position, the mass spectra after irradiation exhibit decreasing signals corresponding to the substrates and the appearance of new peaks corresponding to photoproducts. In the mass spectra of PdOEP derivatives irradiated in the presence of oxygen, starting from a derivative containing one phenyl group substituted at the *meso* position (PdOEP1, *n* = 1, Figures S8 and S9), the formation of a product with a mass higher by 32 *m*/*z* than that of the initial compound can be observed. Other compounds exhibit similar behavior (*n* = 2, 3, 4; Figures S10, S11, and S14–S18). An exception within this series is PdOEP2c (Figures S12 and S13), for which the precise identification of the oxygenated photoproduct remains inconclusive. This compound shows the highest photostability among the series (Table 1, Φ_pb_ ∼0.8 × 10^–7^). The high photostability resulted in only 15% decomposition of this derivative after 44 h of irradiation (Figure S1). The observed *m*/*z* values assigned to all analyzed substrates and their photoproducts are listed in Table 3.

**Table 3:**
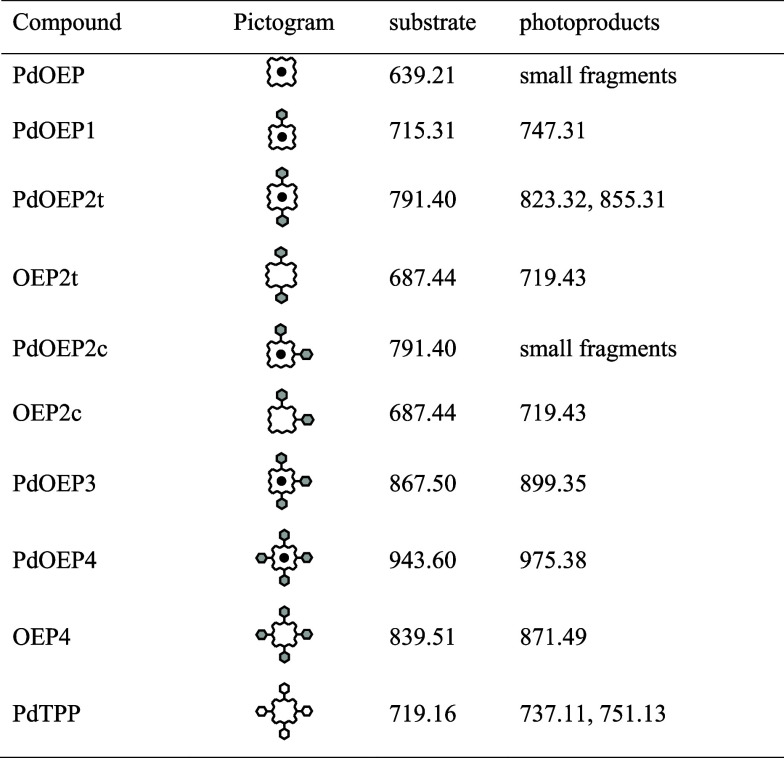
Molar Masses (M, g/mol) of Substrates and Mass to Charge Ratios (*m*/*z*) of Photoproducts Formed under Irradiation of the Studied Porphyrins in Nondegassed Toluene

The mass spectra of free-base OEPt, OEP2c, and OEP4 suggest the formation of a similar photoproduct with a mass 32 *m*/*z* higher than that of the initial compound (Figures S20–S25).

### 3.4 Electrochemistry

The typical voltammograms obtained for all investigated compounds (Figure S26) demonstrate a similar behavior characteristic for a reversible one-electron transfer. For a reversible redox reaction, the potentials *E* ^ox^ and *E* ^red^ can be represented by the half-wave potentials *E*_1/2_^ox^ and *E*_1/2_^red^. The half-wave potentials are calculated according to the equation

$$E_{1/2}=1/2\times \left(Epc+Epa\right)$$

where *E*_pc_ and *E*_pa_ are the cathodic and anodic peak potentials, respectively.^13,14^

The results of the electrochemical measurements are summarized in Table 4. The difference between the two half-wave potentials (*E*_1/2_^ox^–*E*_1/2_^red^) measured for the reference metallocomplexes PdOEP and PdTPP agrees well with the literature data (Table 4). The lowest oxidation potential, 0.35 V, was observed for PdOEP4, whereas the highest potential, 0.76 V, was recorded for PdTPP.

**Table 4:** Summary of Half-Wave Redox Potentials for a Series of PdOEP Derivatives and PdTPP^a^

compound	*E*_1/2_^red^	*E*_1/2_^ox^	*E*_1/2_^ox^–*E*_1/2_^red^	*E*_T_	*E_1/2_^red^	*E_1/2_^ox^	HOMO^b^	LUMO^b^
PdOEP	–1.98 (1)	0.41 (1)	2.39	1.89	–0.09	–1.48	–5.11	–2.09
			2.35^c^					
PdOEP1	–2.02 (1)	0.46 (1)	2.48	1.88	–0.14	–1.42	–5.09	–2.09
PdOEP2t	–2.00 (1)	0.50 (1)	2.50				–5.08	–2.09
PdOEP2c	–2.00 (1)	0.46 (1)	2.46	1.85	–0.15	–1.39	–5.07	–2.11
PdOEP3		0.48 (1)		1.80	-	–1.32	–5.05	–2.12
PdOEP4	–2.14 (1)	0.35 (1)	2.49	1.76	–0.38	–1.41	–5.04	–2.14
PdTPP	–1.80 (1)	0.76 (1)	2.56	1.80	0.00	–1.04	–5.34	–2.35
			2.46^d^					

a Potentials (*E*_1/2_) are given vs Fc/Fc^+^ in [V]. Triplet energy (*E*_T,_ [eV]) and estimated excited-state redox potentials (*E_red_ and *E_ox_) in [V]. Molecular orbitals energy^b^in [eV].

b From DFT calculations using the B3LYP functional and def2-SVP basis set.

c Ref 26.

d Ref 27.

## 4 Discussion

Photodegradation of Zn and Mg derivatives of tetraphenylporphyrin (TPP) in nondegassed solvent provides an open-chain biladienone complex.^15–18^ The photoproduct of photoreaction of Zn and Mg derivatives of octaethylporphyrin is formylbiliverdin,^19^ which is structurally similar to biladienone. Biladienone (or formylbiliverdin) is an open-chain molecule formed as a result of breaking the C_*m*_–C_α_ bond in porphyrin and attaching two additional oxygen atoms. The open-chain structure is stabilized by the heavy atom in the center of the macrocycle. It partially retains the structure of the porphyrin (Figure 3c). Analysis of changes induced by light irradiation in mass spectra and electronic absorption spectra allows the assignment of photoproducts for the investigated series of PdOEP derivatives. Mass spectra indicate that the stable photoproduct in toluene has a mass 32 *m*/*z* greater than the mass of the substrate (Table 3). The electronic absorption spectra of the studied systems indicate the rise of two broad bands of the photoproduct: one is starting at 800 nm and the other appears below 650 nm. Such behavior is similar to previously reported results of photodestruction of Mg and Zn derivatives of TPP and OEP.^15,16^ It obviously suggests that the stable photoproduct (starting from the derivative with one phenyl through successive derivatives, including also PdTPP) corresponds to a derivative of biladienone (formylbiliverdin) (Figure 3c).

**Figure 3:**
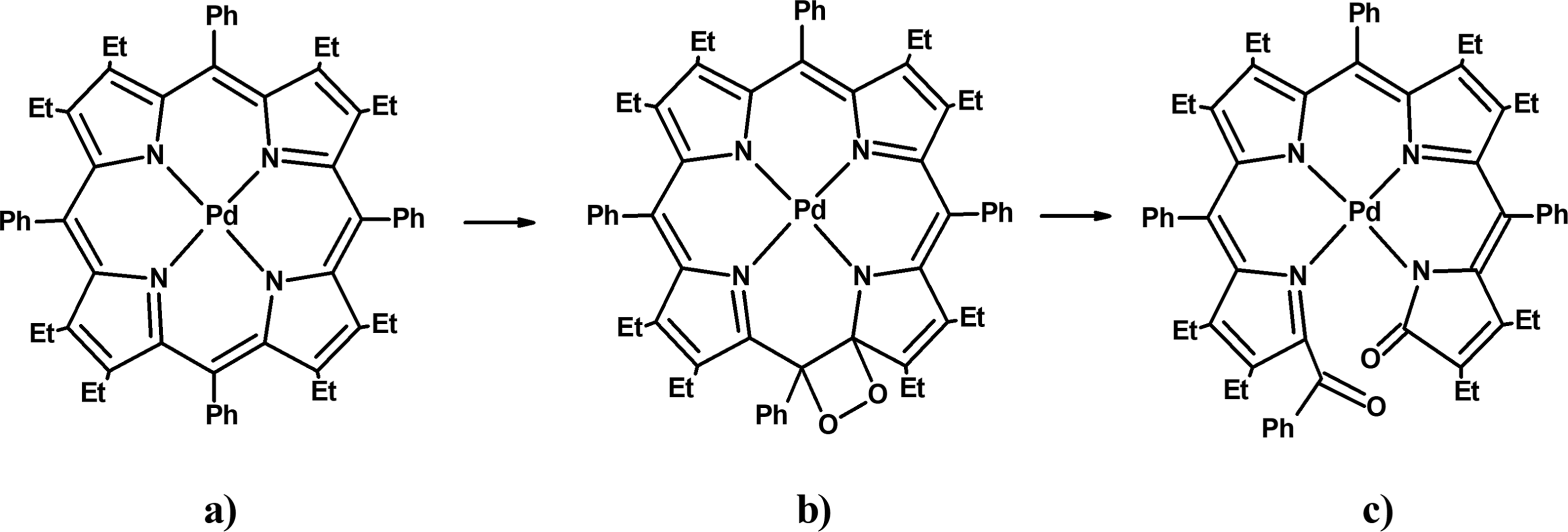
PdOEP4 (a), intermediate product (b), and product of photoreaction—derivative of biladienone (c).

The quantum yields of photodegradation of PdOEP (1.5 × 10^–7^) and PdTPP (4.5 × 10^–7^) considered as reference compounds here are significantly lower than those of, e.g., ZnTPP (8.1 × 10^–6^, ref 15) or ZnOEP (2 × 10^–5^, unpublished results). This fact can be explained by the higher electronegativity of the Pd atom and, as a result, an overall higher oxidation potential of palladium porphyrins.^18^ As previously mentioned, among the investigated complexes, PdOEP2c exhibits the highest photostability under aerobic conditions (Table 1). The triplet lifetime of this compound is 60 ns, which is extremely short for porphyrin derivatives (Table 2). This short lifetime is attributed to steric hindrance in the molecule between the phenyl and ethyl groups, leading to a nonplanar structure of the porphyrin macrocycle and a rapid quenching of the triplet state.^8,20^ Two structures with slightly lower photostability are PdOEP3 and PdOEP4, characterized by triplet lifetimes of 48 and 30 ns, respectively (Table 2). Shorter triplet lifetimes should lead to higher photostability in triplet state photoreactions. However, with the growing number of phenyl substituents with a slightly electron-donating character,^21,22^ a compound becomes more susceptible to attack by singlet oxygen leading to the oxidation under light irradiation. This finding is confirmed by calculations of HOMO energies in the triplet state for PdOEP3 and PdOEP4, which are destabilized compared to those of PdOEP2c (Figure S27).

Table 1 shows that the photobleaching rate constant (*k*_pb_ = Φ_pb_/(Φ_T_×τ_*T*_), where τ_*T*_ is the triplet lifetime and Φ_*T*_ is the triplet state formation yield) increases approximately 6 times when passing from PdOEP2c to PdOEP4. PdOEP4 exhibits the highest rate of photobleaching among all considered metallocomplexes. Nevertheless, due to the shortest triplet lifetime, the photostability of PdOEP4 is only three times lower than that of the most stable PdOEP2c. These two parameters (triplet lifetime and the number of phenyl substituents) act on photostability in opposite directions.

Under aerobic conditions, PdOEP1, PdOEP2t, and PdTPP are the least photostable compounds with triplet lifetimes of 128, 130, and 347 ns, respectively (Table 2). As was previously shown, the porphyrin macrocycle of derivatives substituted by one phenyl (PdOEP1) or two phenyls at positions 5 and 15 (PdOEP2t) is only slightly distorted from planarity.^22^ The more planar structures are characterized by a longer triplet lifetime and, as a result, lower photostability. The reference compound, PdOEP, stands out in the studied series under aerobic conditions, exhibiting photostability (photodestruction quantum yield, Φ_pb_ of 1.5 × 10^–7^) comparable to the most photostable PdOEP2c (Φ_pb_ of 0.8 × 10^–7^) (Table 1). The lack of phenyl substituents in PdOEP likely hinders the cleavage of the methine bridges. The photodegradation rate of PdOEP is 0.62 s^–1^, which is the lowest value in the entire series.

To explain these findings, oxidation potentials in the excited states (^*^ *E*_1/2_^red^ = *E*_1/2_^red^ + ^*^ *E*_0,0_, ^*^ *E*_1/2_^ox^ = *E*_1/2_^ox^ – ^*^ *E*_0,0_, ref 13) of the investigated complexes were calculated and are summarized in Table 4. The triplet state energies of the investigated complexes used in the calculations were obtained previously.^8^ It should be noted that complexes with a more positive value of ^*^ *E*_1/2_^red^ have a stronger photooxidizing power, whereas those with a more negative value of ^*^ *E*_1/2_^ox^ exhibit stronger photoreducing properties.

There is no direct correlation between the photostability of the investigated complexes and the excited-state oxidation potentials. For example, the excited-state oxidation potential of PdOEP is more negative (−1.48 V, Table 4) than that of the entire series, and this compound should be the easiest to oxidize in the series, which is not the case. Despite its long triplet lifetime (241 ns, Table 2), PdOEP exhibits one of the highest photostabilities in nondegassed toluene. Additionally, the identification of photoproducts based on mass spectrometry analysis was unsuccessful. This compound likely undergoes decomposition into smaller fragments, bypassing the biladienone product. However, a definitive identification of the resulting photoproducts would require further studies.

Omitting PdOEP, where the postulated decomposition pathway differs from the rest of the series, the most photostable compound, PdOEP2c, exhibits an excited-state oxidation potential of −1.39 V, which is close to the potential of PdOEP1 (−1.42 V), the least photostable complex among the investigated compounds. At this moment, it is difficult to explain the lack of a direct correlation between photostability and excited-state oxidation potentials, and additional studies are required.

To clarify the heavy atom effect and the role of nonplanarity in the porphyrin macrocycle, we expanded the study by investigating the photostability of three free-base porphyrins: OEP2t, OEP2c, and OEP4. We also compared their photostability to the already known photostabilities of TPP and OEP.

According to the literature, OEP2t, OEP2c, and OEP4 exhibit a dynamic nonplanarity in their first excited triplet states due to steric interactions between the *meso*-phenyl groups and the bulky ethyl groups at the β-pyrrolic positions. The effect significantly alters the triplet state geometry and is responsible for shortening of the triplet state lifetime.^23,24^

The photostability of these three free-base porphyrins was found to be 7-fold, 300-fold, and 5500-fold lower, respectively, than that of their corresponding palladium metallocomplexes. This significant decrease in photostability aligns well with quantum-chemical calculations (DFT, TD-DFT, and B3LYP/def2SVP) of optimized molecular geometries for the first excited triplet state (T_1_) and the energies of the frontier occupied molecular orbitals. The introduction of a palladium ion at the center of the pyrrolic macrocycle reduces the nonplanarity of the porphyrin ring in the T_1_ state. To visualize these structural changes in the T_1_ state upon transition from free-base nonplanar porphyrin (OEP2t, OEP2c, and OEP4) to their Pd metallocomplexes, we calculated the dihedral angle (C_α_–C_m_–C_α_–N, Figure 4), which illustrates the deviation of the pyrrole ring (adjacent to the bulky substituents) from the plane of the porphyrin macrocycle due to steric effects. The dihedral angle changes only slightly between the OEP2t and PdOEP2t, from 10° to 9°. However, in the significantly more nonplanar OEP2c and OEP4, the dihedral angle decreases from 58° to 18° and from 45° to 25° upon metalation, respectively (Table 5). The deviation from planarity also significantly alters the C_m_–C_α_ (Figure 4) bond length within the porphyrin core, which appears to be crucial in the discussed photoreaction. The C_m_–C_α_ bond lengths increase for the free-base derivatives compared to their Pd complexes (Table 5). These structural changes further influence the energies of the highest occupied molecular orbitals (HOMOs) of the OEP2t, the OEP2c, and the OEP4 free-base porphyrins. The HOMO energies are 0.23, 0.62, and 0.58 eV higher than those of their corresponding Pd derivatives (Table 5 and Figure 4). The destabilization of the HOMO of nonplanar, free-base porphyrins leads to lower oxidation potentials, ultimately resulting in reduced photostability.

**Figure 4:**
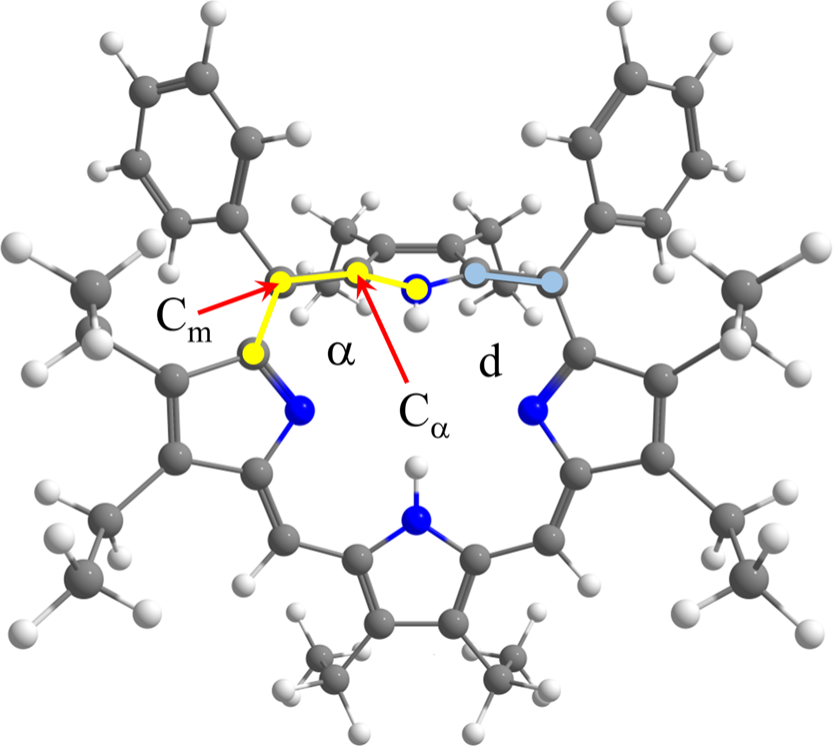
Dihedral angle, α (C_α_–C_m_–C_α_–N, atoms marked by yellow circles), representing the degree of nonplanarity of the T_1_ state of OEP2c, and bond length, d (C_α_–C_m_, atoms marked by cyan circles).

**Table 5:** Dihedral Angle, α (C_α_–C_m_–C_α_–N), and Bond Length, d (C_α_–C_m_), Representing the Degree of Nonplanarity in the Optimized Geometry of the T_1_ State (TD-DFT and B3LYP/def2SVP) of OEP2t, OEP2c, OEP4, and Their Pd Metallocomplexes

	dihedral angle, α (C_α_–C_m_–C_α_–N) [deg]	bond length, d (C_α_–C_m_) [Å]
OEP2t	10	1.458
OEP2c	58	1.488
OEP4	45	1.473
PdOEP2t	9	1.437
PdOEP2c	18	1.444
PdOEP4	25	1.448

An especially interesting case is that of OEP2c, a compound substituted by two phenyl groups in the neighboring *meso* positions as well as its Pd derivative. OEP2c demonstrates the lowest quantum yield of singlet oxygen generation, close to 2% (Table 1), among all of the free-base porphyrins considered in this study. This finding aligns with the low efficiency of singlet oxygen formation observed for similar compounds reported in the literature.^25^ In reference 25, the authors describe the photophysics of octamethylporphyrins substituted with bulky phenyl groups in the *meso* positions. The study reports that compounds with two neighboring phenyl groups and those with three phenyl groups exhibit a very low quantum yield of singlet oxygen generation (6% and 15%, respectively). In spite of low efficiency of singlet oxygen generation, the rate constant of photodegradation of OEP2c is very high, and only one compound in the series is less stable: OEP4 (Table 1), a derivative substituted with four phenyls. According to calculations, the triplet state geometry of OEP2c is characterized by the highest dihedral angle C_α_–C_m_–C_α_–N (58°) and the largest length of the C_m_–C_α_ bond (1.488 Å). On the other hand, OEP2c has only two sites suitable to be attacked by singlet oxygen, compared to the four positions in OEP4. The geometry of the triplet state of PdOEP2c becomes significantly more planar compared to OEP2c. The dihedral angle decreases to 18°, and the C_m_–C_α_ bond length reduces to 1.44 Å (Table 5). The changes in the geometry of the triplet state of OEP2c upon metalation lead to the stabilization of the HOMO energy. The HOMO energy of PdOEP2c is lower than those of PdOEP3 and PdOEP4 and comparable to those of PdOEP2t and PdOEP1. Several factors, such as the stabilization of HOMO energies upon metalation, a short triplet lifetime due to the heavy atom effect, and only two sites available for attack by singlet oxygen, contribute to the highest photostability of PdOEP2c within the entire series of investigated compounds.

Under anaerobic conditions, the photostability of the investigated systems undergoes significant changes. The photostability of OEP and TPP as well as their metallocomplexes PdOEP and PdTPP decreases significantly (Table 1, photodestruction quantum yield within the range of 3 × 10^–5^ for PdOEP and 4.4 × 10^–6^ for OEP). The triplet states of these compounds in deoxygenated toluene are hundreds of microseconds (Table 2). Therefore, the decrease in photostability under anaerobic conditions (oxygen being a strong quencher of triplet states) is associated with triplet lifetimes increasing by about 3 orders of magnitude. It should be noted that under anaerobic conditions, Pd metallocomplexes of TPP and OEP are significantly less stable than the free-base TPP and OEP. One possible explanation is that, under oxygen-free conditions, the mechanism of photodestruction for palladium metallocomplexes may differ from that of free-base porphyrins.

Conversely, the photostability of PdOEP2c, PdOEP3, and PdOEP4 in oxygen-free conditions increases by 2 orders of magnitude, due to short-lived triplet states and the absence of oxygen which is needed for oxidation reaction (Table 1, photodestruction quantum yield, inversely proportional to photostability, decreases to a value below 10^–9^). The exact value of Φ_pb_ was not determined due to the significantly prolonged duration of the experiment. For example, for PdOEP2c, no changes in electronic absorption spectra were observed even after 60 h of irradiation.

The remaining two compounds, PdOEP1 and PdOEP2t, exhibited from 2- to 5-fold greater photostability (Table 1) with a slight extension of triplet lifetimes (around 200 ns) in degassed solutions. In this case, the significantly lower oxygen concentration is partially balanced by an increasing triplet state lifetime by about 35%.

Changes in the electronic absorption spectra suggest the formation of different photoproducts under anaerobic conditions, but the precise identification requires additional studies. These studies are complicated by the increased photostability under anaerobic conditions, which significantly prolongs the experimental time.

## 5 Conclusions

The quantum yields of photodestruction were determined for a series of free-base and palladium octaethylporphyrins in nondegassed and deaerated toluene. Due to the heavy atom effect and the high efficiency of the triplet state formation, it seems safe to conclude that the photoreaction occurs in the triplet state.

One of the reference compound—PdOEP—is characterized by high photostability under atmospheric conditions (Φ_pho_ = 1.5 × 10^–7^, Table 1), especially compared to well-known Zn or Mg derivatives. This compound likely undergoes decomposition into smaller fragments during the photoreaction. A definitive determination of the resulting photoproducts requires additional studies. The photostability of PdOEP under anaerobic conditions decreases by 2 orders of magnitude, as the triplet lifetime, due to the lack (or very weak) of quenching by molecular oxygen, increases by nearly 3 orders of magnitude.

The other reference compound, PdTPP irradiated under atmospheric conditions, is approximately 2.5 times less photostable than PdOEP. The photoproduct formed during irradiation results from the cleavage of the porphyrin C_m_–C_α_ bond and the attachment of two additional oxygen atoms. The photostability of PdTPP decreases upon deoxygenation, as the triplet lifetime extends by 3 orders of magnitude. The rate constants of the photobleaching reaction of PdTPP and PdOEP in degassed toluene are very similar, 0.09 and 0.11 s^–1^, respectively.

In the case of porphyrins with the number of phenyl groups increasing from 1 to 4, illuminated under aerobic conditions, a photoproduct containing two oxygen atoms at the intersection of the porphyrin C_m_–C_α_ bond likely forms. The resulting toluene-stable photoproduct is assigned to the palladium derivative of biladienone (Figure 3c). The highest photostability in nondegassed toluene in the series is exhibited by PdOEP2c where the porphyrin moiety is disubstituted with phenyls in positions 5 and 10. Photostabilities of PdOEP2c, PdOEP3, and PdOEP4 under anaerobic conditions are extremely high (Φ_pb_ < 10^–9^) due to the lack of oxygen necessary for the photoreaction and the inherent properties of the investigated systems (short triplet lifetimes). These compounds combine the thermodynamic enhancement of photostability due to large electronegativity of palladium with the kinetic factor, the improvement obtained by shortening the triplet lifetime. The latter seems to be the crucial parameter that determines the photostability, especially under oxygen-free conditions. Another important factor is the nature of the substituent: an electron-withdrawing one should increase photostability or whether an electron-donating one acts in the opposite direction.

In contrast, the photostability of nonplanar free-base porphyrins—OEP2t, OEP2c, and OEP4—is low due to significant changes in the geometry of the first excited triplet state. These changes lead to the destabilization of the energy of the highest occupied molecular orbital and, as a result, a decrease in the oxidation potentials, consequently reducing the photostability.

The effect of nonplanarity on the photostability of porphyrins seems to be an interesting subject for further studies in the context of the photostability of nonplanar porphyrin derivatives in biological systems.

## Supplementary Material



## Data Availability

Data for this article are available at RepOD at https://doi.org/10.18150/H7WWLH.

## Notes

Photostability, mass spectrometry, and electrochemistry data, along with the results of quantum-chemical calculations and photophysical characteristics (PDF)
