# Supplementary material for: Enhancing Photostability by Thermodynamic and Kinetic Factors: Free-Base and Palladium meso-Aryl-octaethylporphyrins
Source: J Phys Chem B. 2025 May 28;129(22):5491–500. doi: 10.1021/acs.jpcb.5c02423 (PMC12147201; doi:10.1021/acs.jpcb.5c02423)
Supplement: Supplementary file 1 [file jp5c02423_si_001.pdf]

Supporting Information for

Enhancing Photostability by Thermodynamic and Kinetic Factors: Free-Base and Palladium *meso*-aryl-octaethylporphyrins

Maciej Banaszek,[1] Barbara Golec,[1,2] Renata Rybakiewicz-Sekita,[1] Jarosław Kowalski,[1] Piotr Szczodry[1] Natalia Dutkiewicz,[2] Jacek Waluk,[1,2] Aleksander Gorski*[2]

[1] *Faculty of Mathematics and Science, Cardinal Stefan Wyszyński University, Dewajtis 5, 01-815 Warsaw, Poland*

[2] *Institute of Physical Chemistry, Polish Academy of Sciences, Kasprzaka 44/52, 01-224 Warsaw, Poland*

Correspondence to: Aleksander Gorski: agorski@ichf.edu.pl

Table of contents

Photostability 2

Mass spectrometry 8

Electrochemistry 18

Quantum-chemical calculations 22

Photophysical characteristics 26

Photostability

Table S1. Spectral changes in absorption observed for non-degassed toluene porphyrin samples as a result of UV irradiation

Compound	Pictogram	Appearing new bands, nm	Disappearing,[a] nm
PdOEP	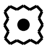	< 300, 577, 600	394, 514, 546
PdOEP1	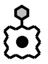	300, 462, 603, 645, > 800	402, 416, 550, 573,[b] 628[b]
PdOEP2t	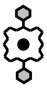	463, 590, 640, 800	405, 518, 550
PdOEP2c	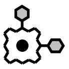	460, 590, 610, 660, > 780 (weak)	413, 526, 560
PdOEP3	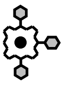	318, 472, 605, 660, > 800	424, 536, 570
PdOEP4	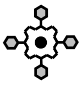	320, 489, 635, > 800	434, 545, 580
PdTPP	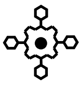	324, 470, 580, 640, > 800	418, 524, 554, 603[b]

[a]Main bands of initial substrates disappearing under irradiation, [b] small bands due to impurities disappearing in addition to main bands of initial substrates

PdOEP

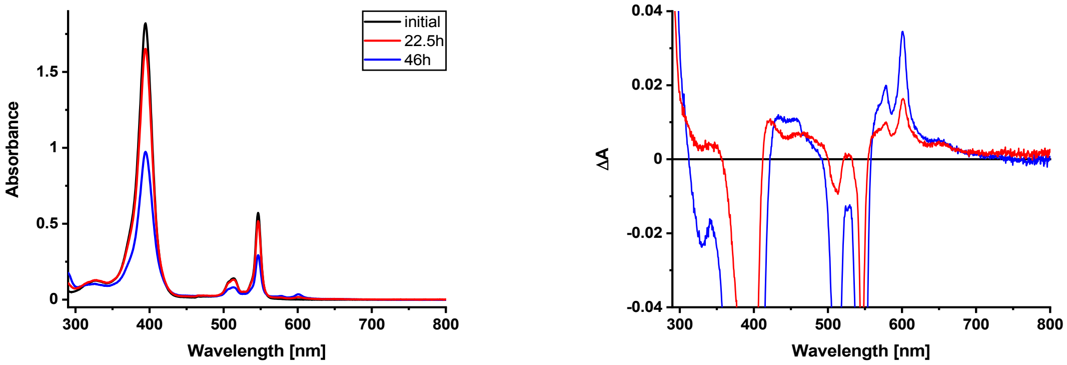

PdOEP1

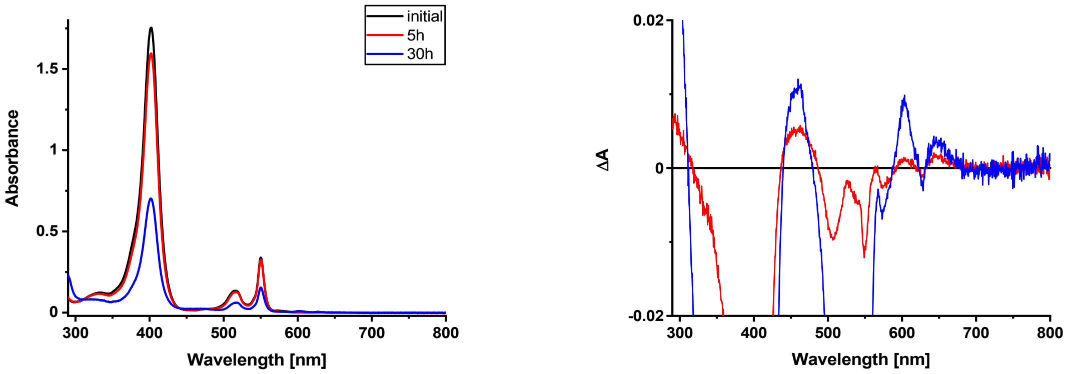

PdOEP2t

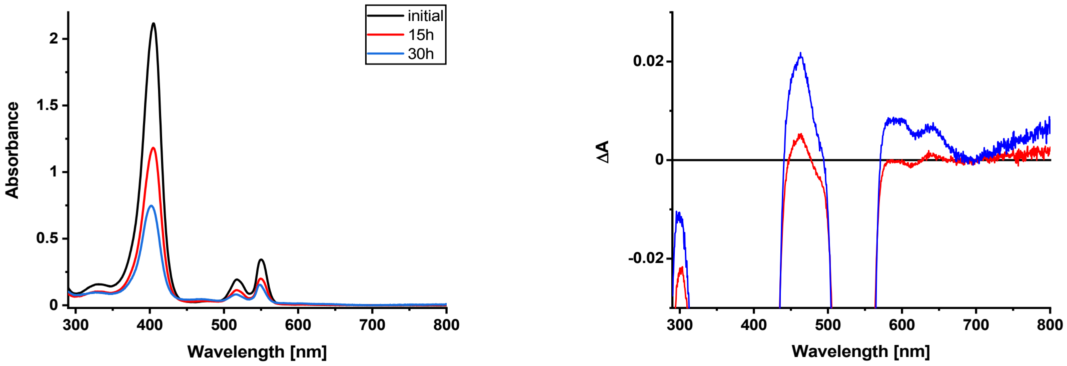

PdOEP2c

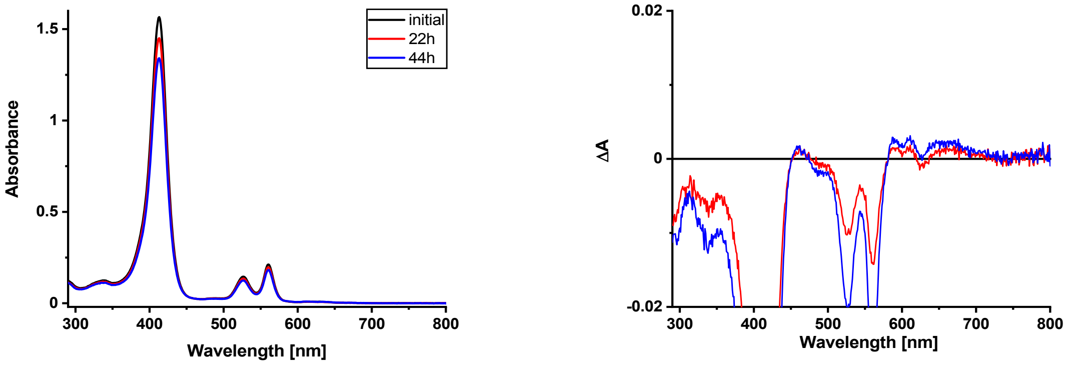

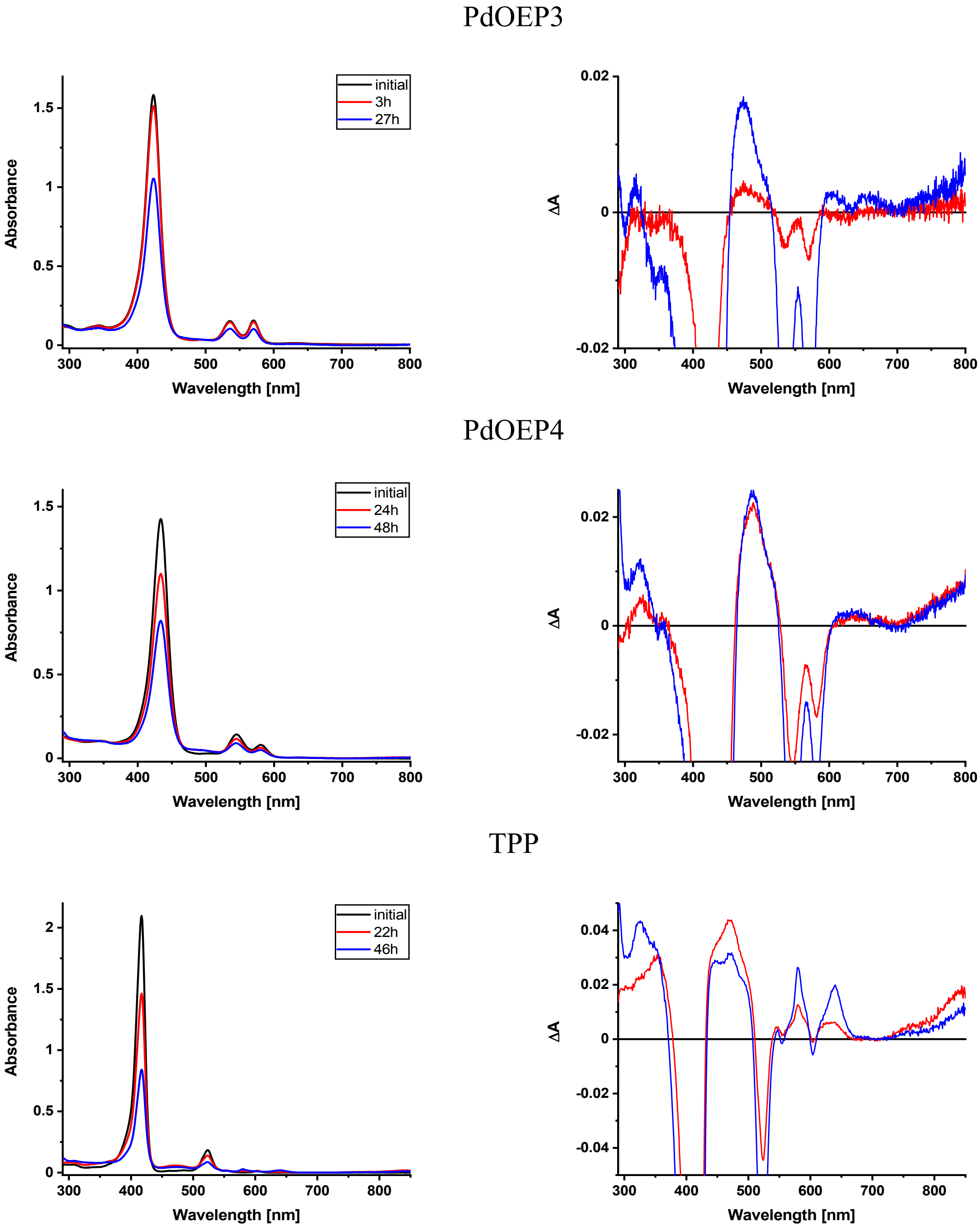

Figure S1. Absorption spectra in non-degassed toluene. Samples were irradiated with two LEDs (385 nm maximum, power of 102 mW for PdOEP and 420 nm maximum, power of 170 mW for the rest of compounds). Left, changes in the absorption spectra. Right, absorption spectra obtained after subtracting the contribution from the substrate.

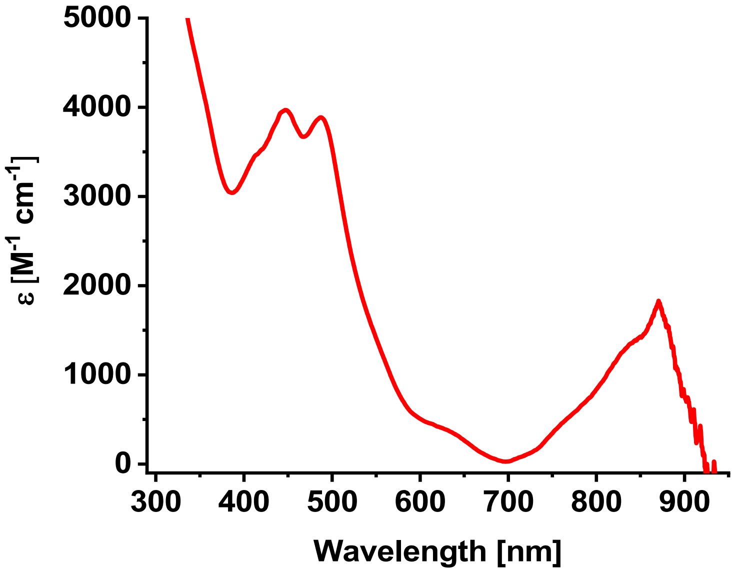

Figure S2. Electronic absorption spectrum of the photoproduct obtained after 23 h of irradiation of PdOEP4 ($c = 6.5 \times 10^{-6}$ M) in toluene at 420 nm (170 mW)

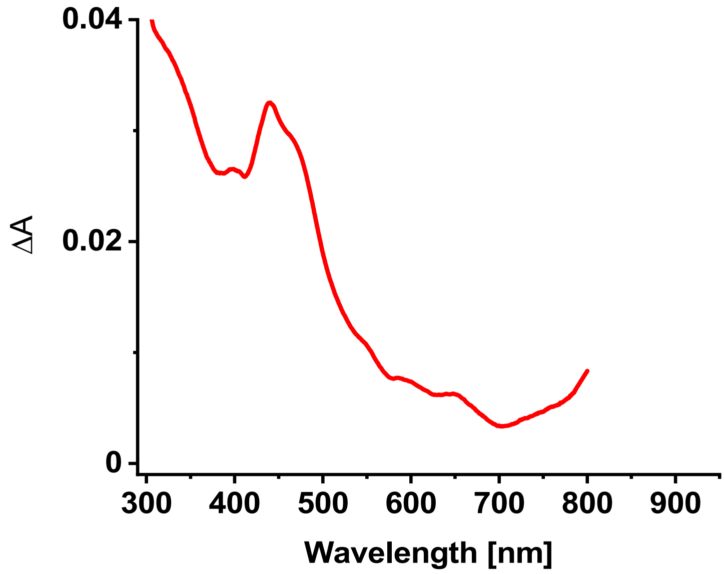

Figure S3. Electronic absorption spectrum of the photoproduct obtained after 27 h of irradiation of PdOEP3 ($c = 9.4 \times 10^{-6}$ M) in toluene at 420 nm (170 mW)

PdOEP

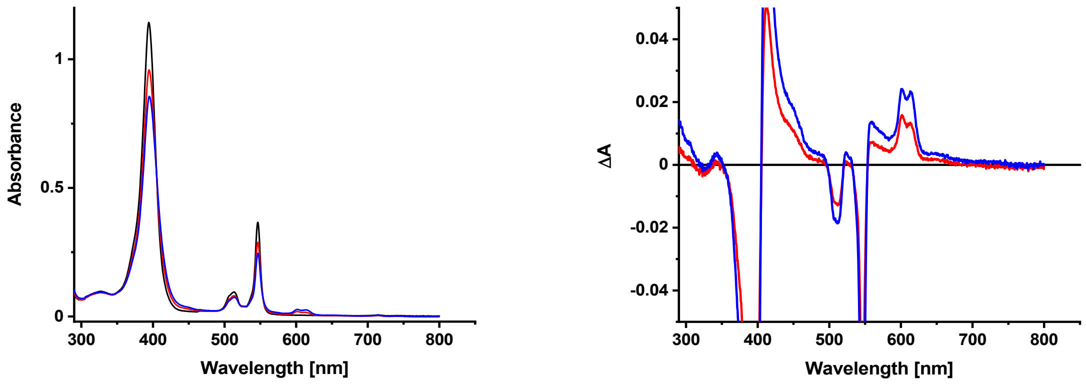

PdOEP2t

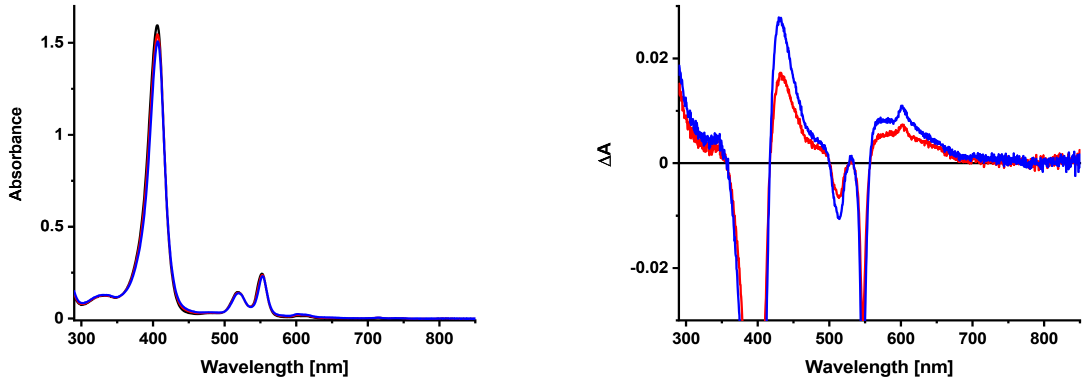

PdTPP

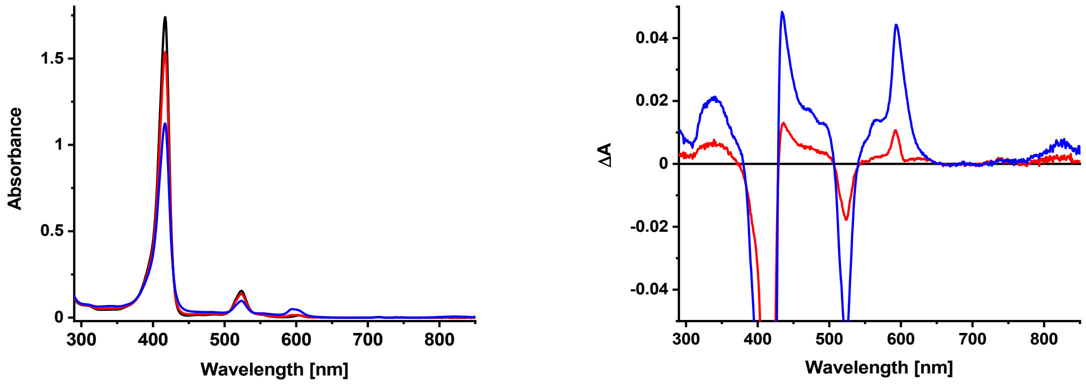

Figure S4. Absorption spectra in deaerated toluene. Samples were irradiated with two LEDs (385 nm maximum, power of 102 mW for PdOEP and 420 nm maximum, power of 170 mW for the rest of compounds). Left, changes in the absorption spectra. Right, absorption spectra obtained after subtracting the contribution from the substrate.

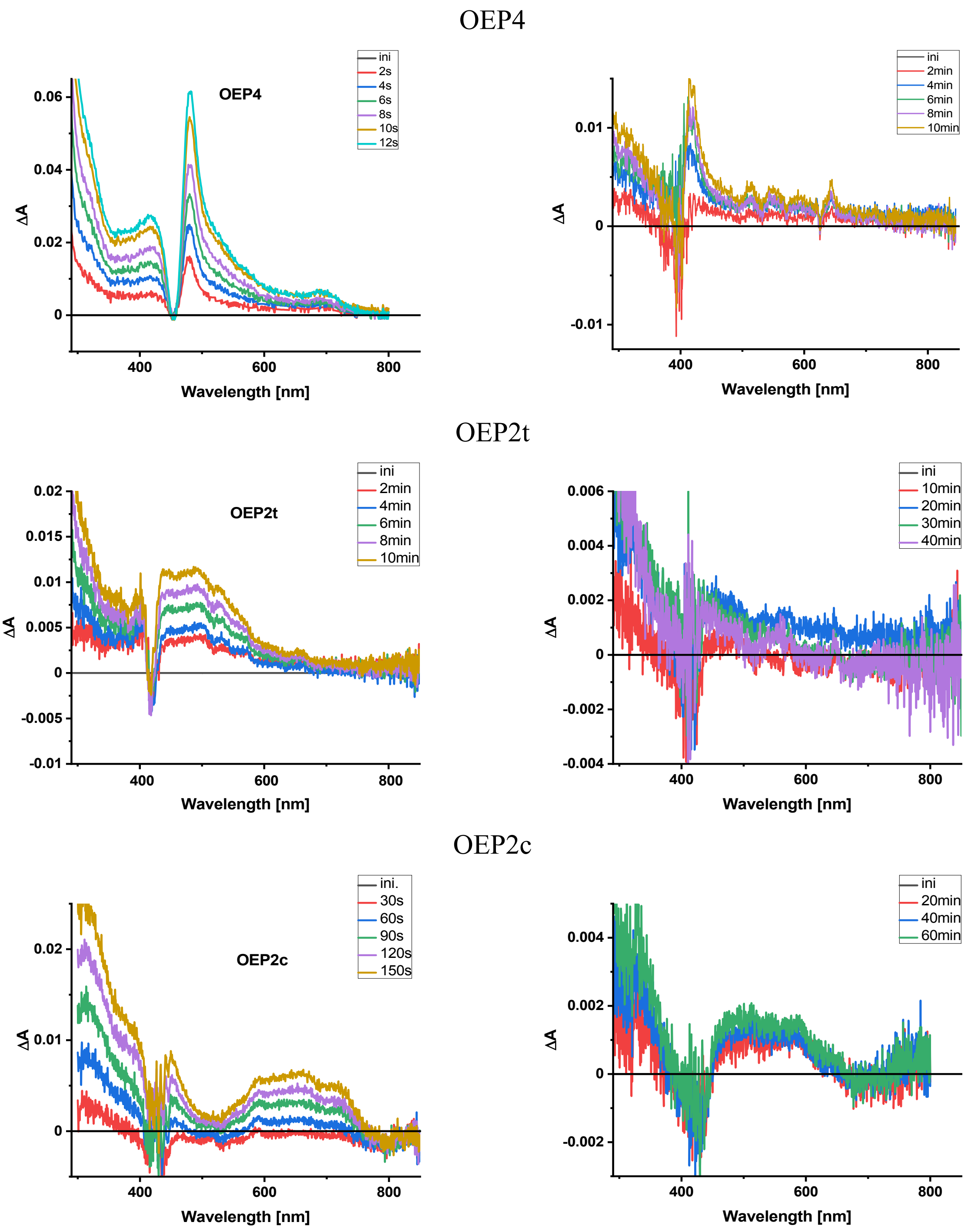

Figure S5. Absorption spectra of OEP4, OEP2t, and OEP2c (from top to bottom) after irradiation, obtained after subtracting the contribution from the substrate in non-degassed toluene (left) and deaerated toluene (right).

Mass spectrometry

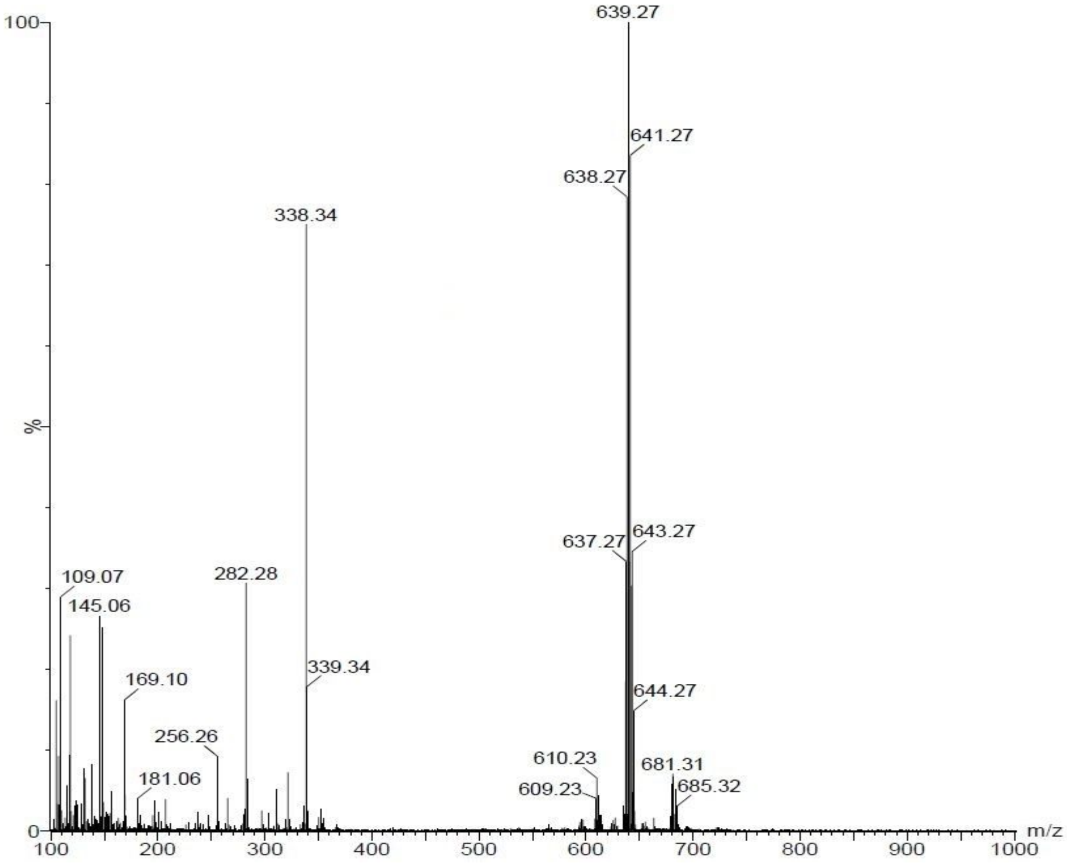

Figure S6. Mass spectrum of PdOEP before irradiation.

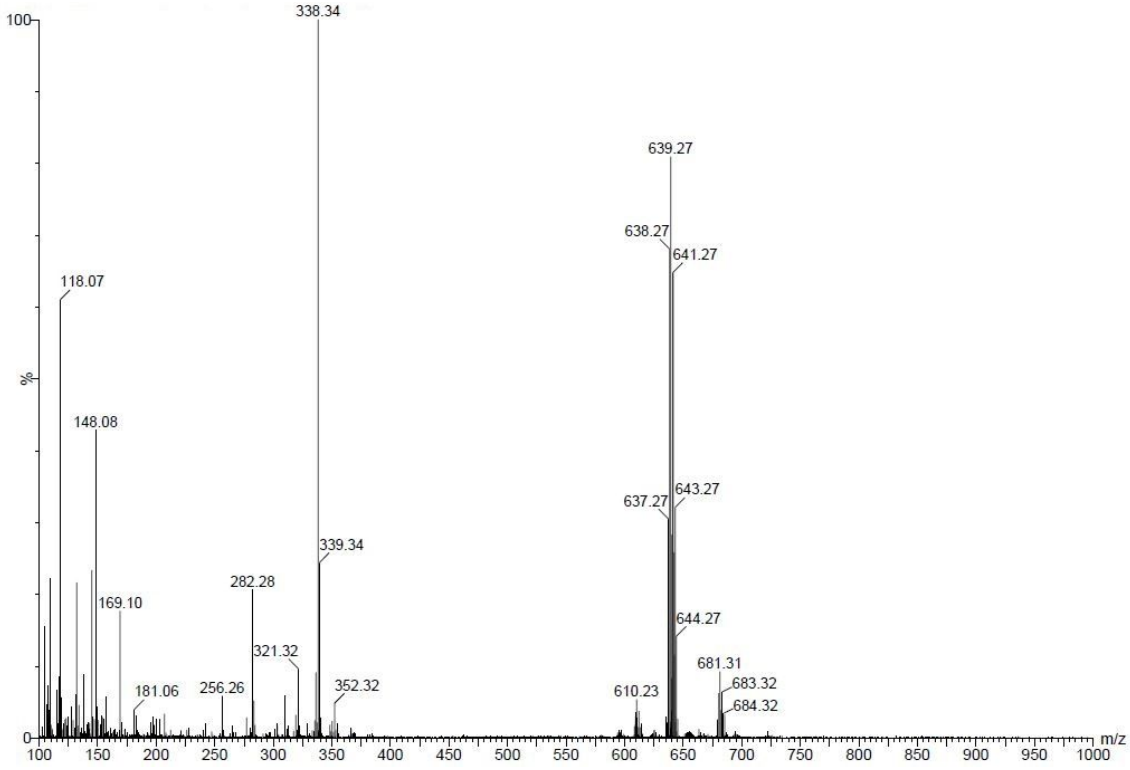

Figure S7. Mass spectrum of PdOEP after irradiation

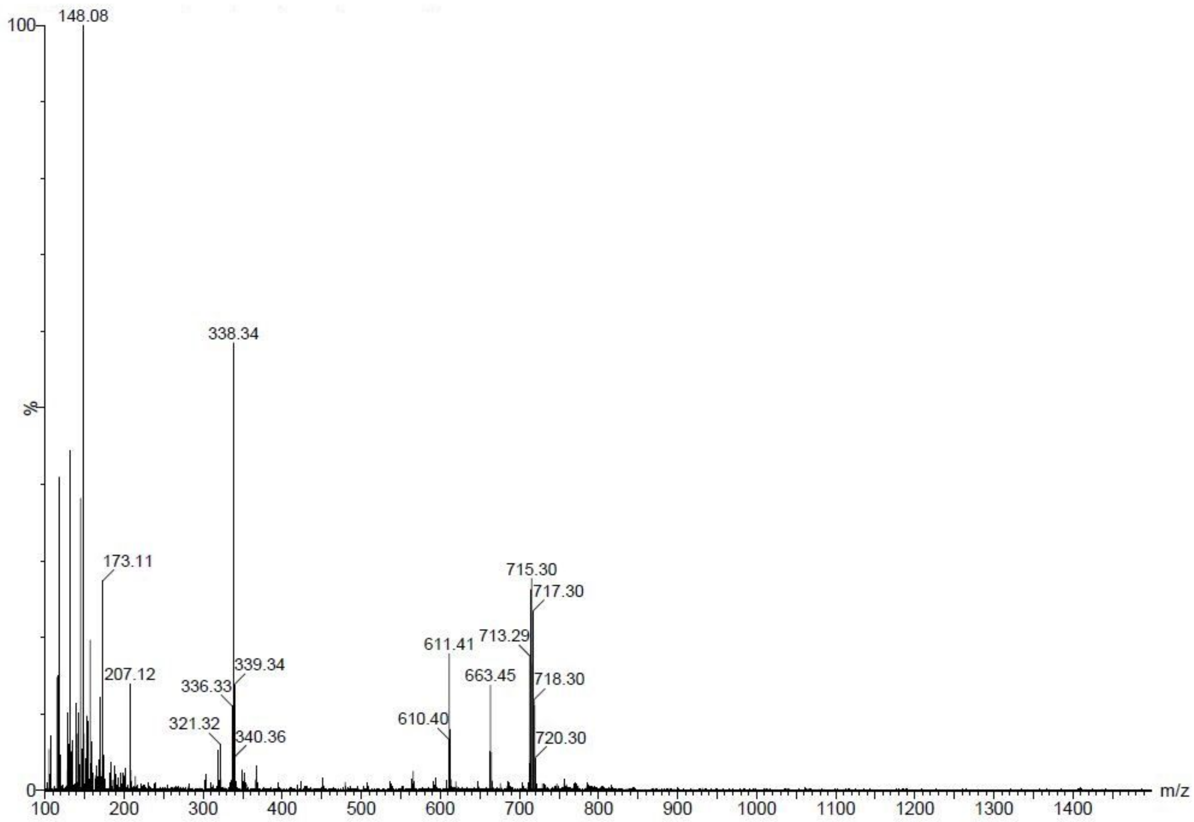

Figure S8. Mass spectrum of PdOEP1 before irradiation

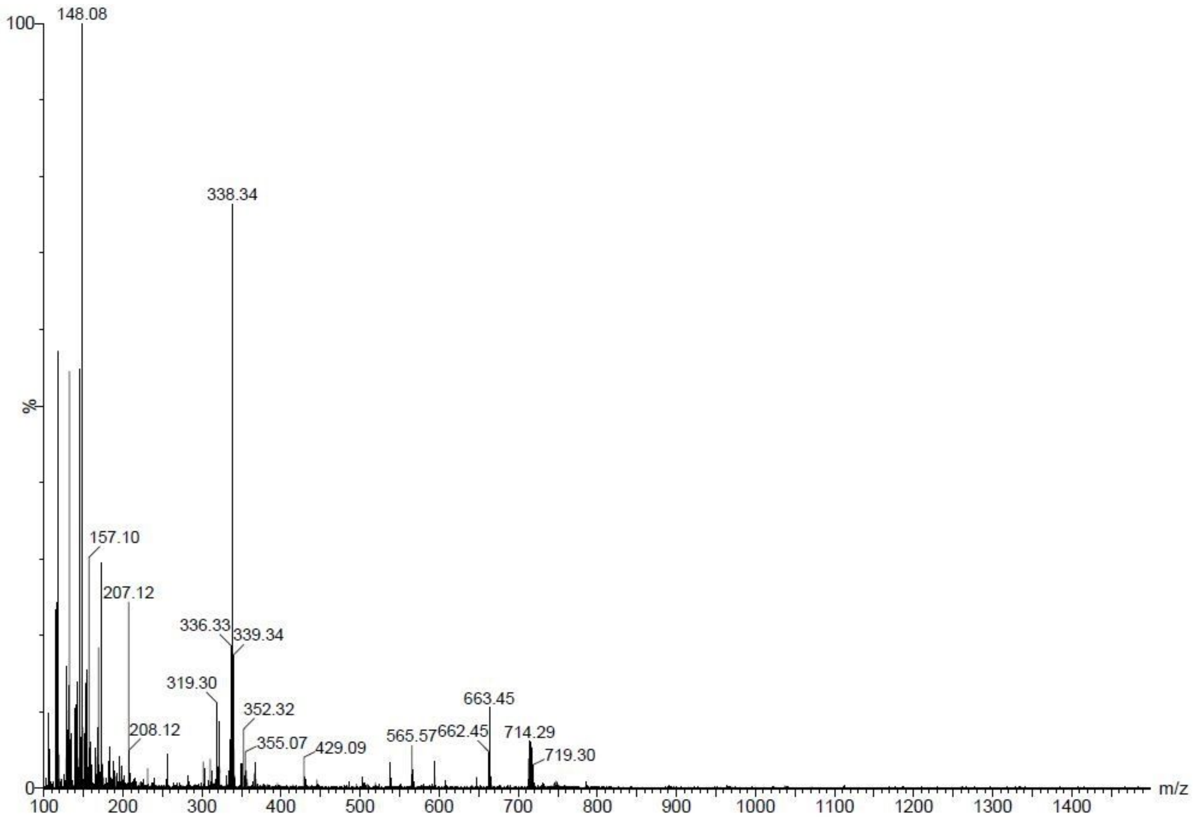

Figure S9. Mass spectrum of PdOEP1 after irradiation

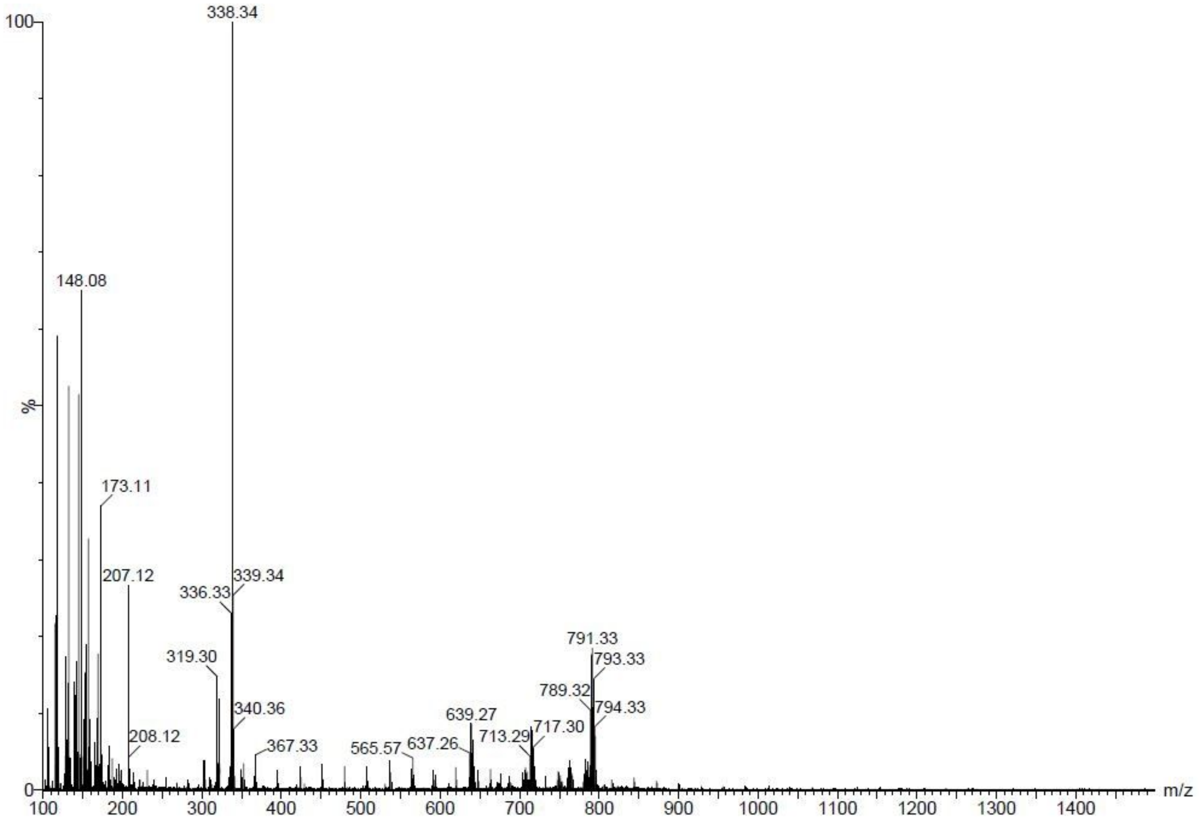

Figure S10. Mass spectrum of PdOEP2t before irradiation

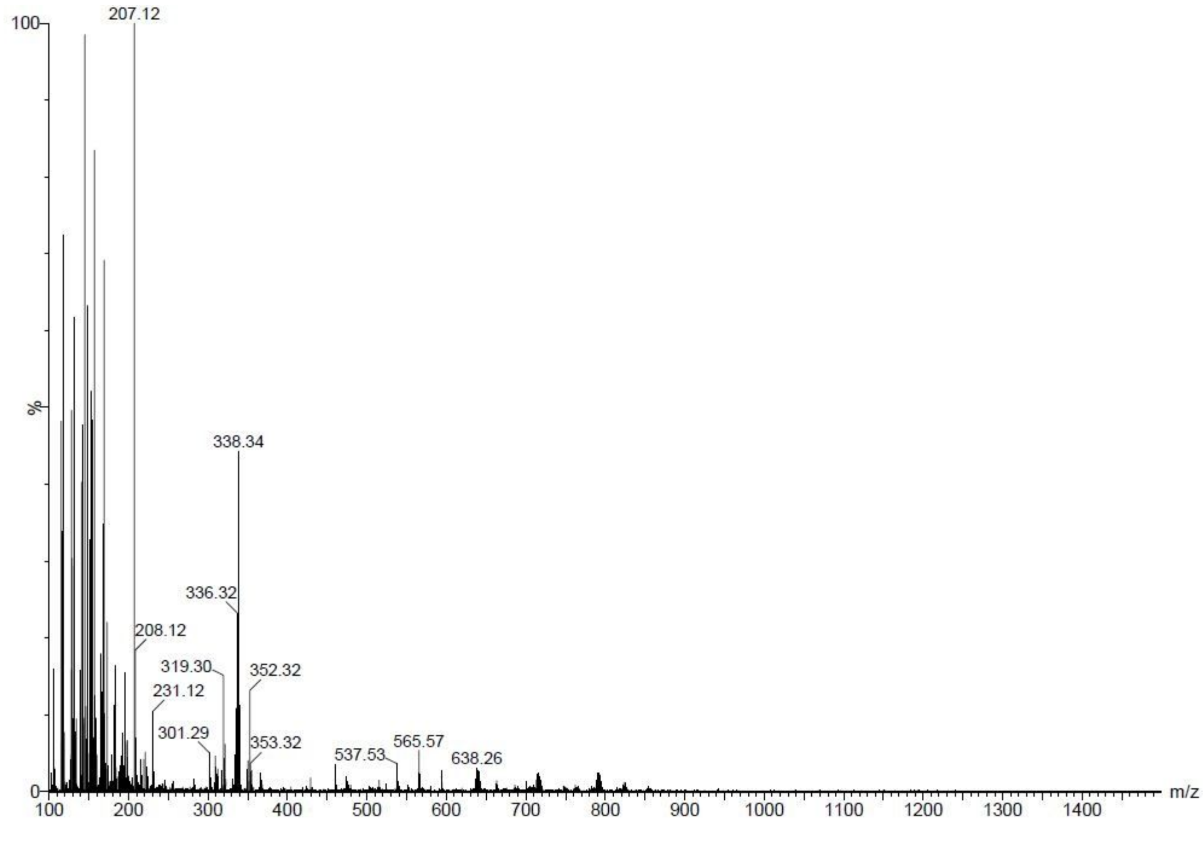

Figure S11. Mass spectrum of PdOEP2t after irradiation

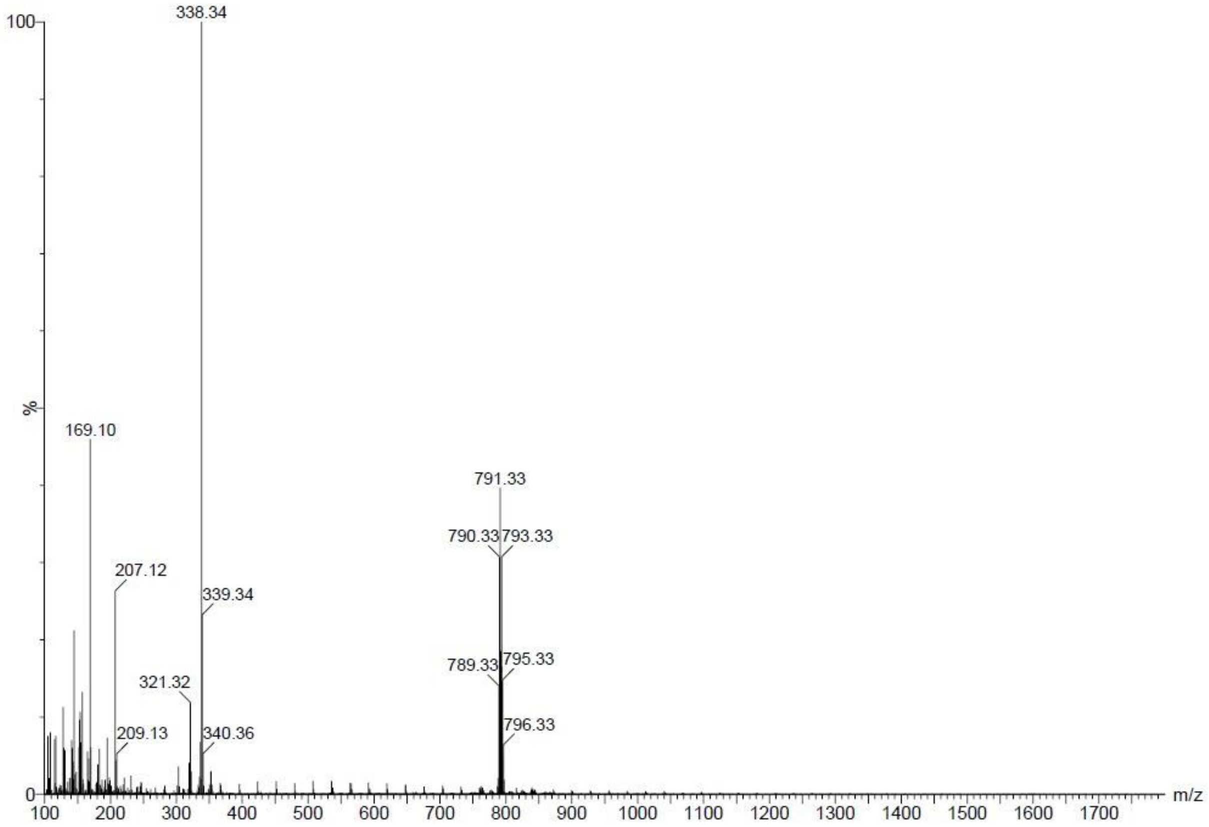

Figure S12. Mass spectrum of PdOEP2c before irradiation

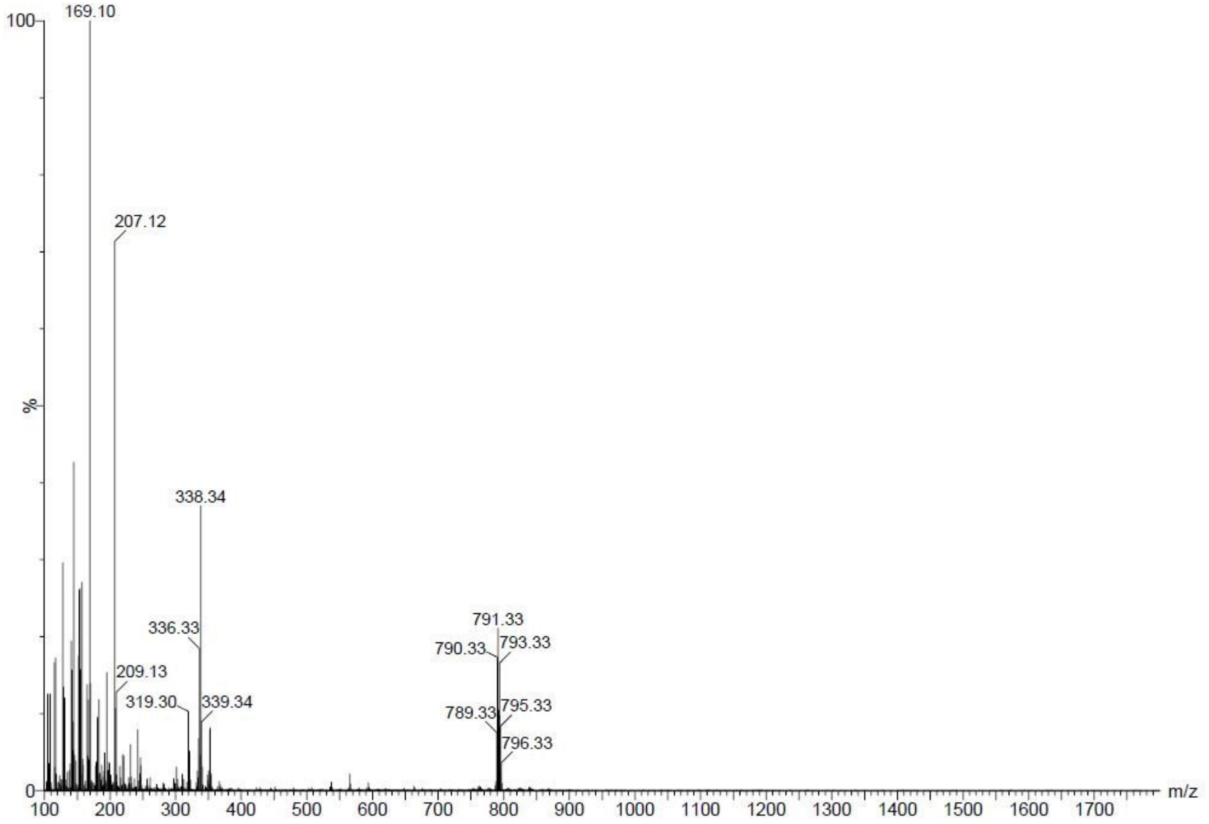

Figure S13. Mass spectrum of PdOEP2c after irradiation

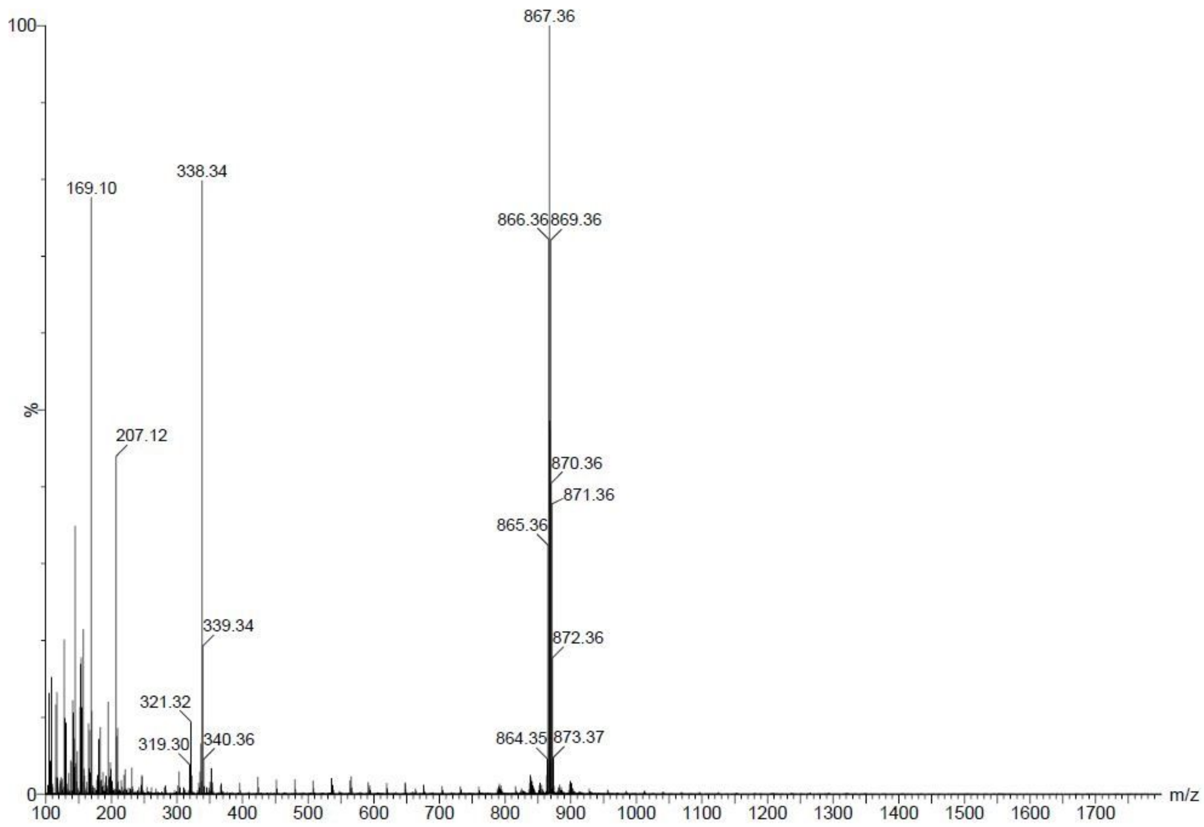

Figure S14. Mass spectrum of PdOEP3 before irradiation

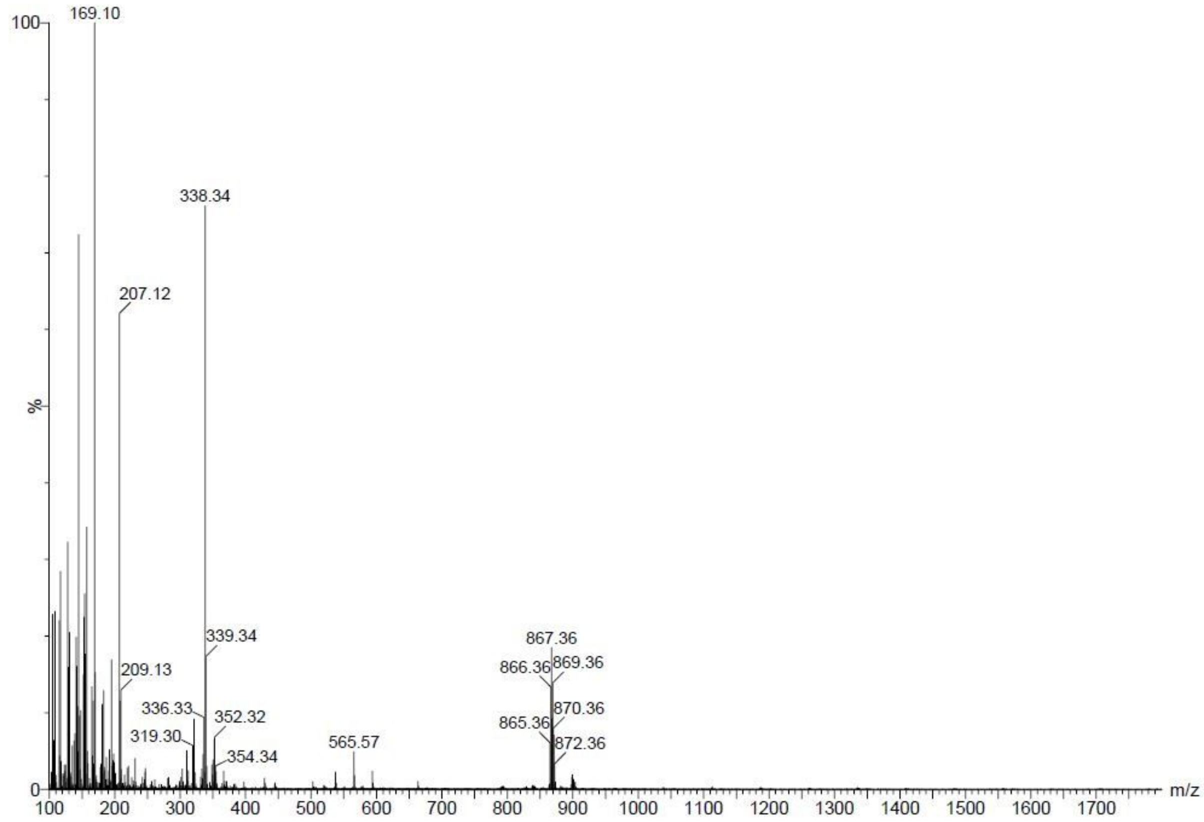

Figure S15. Mass spectrum of PdOEP3 after irradiation

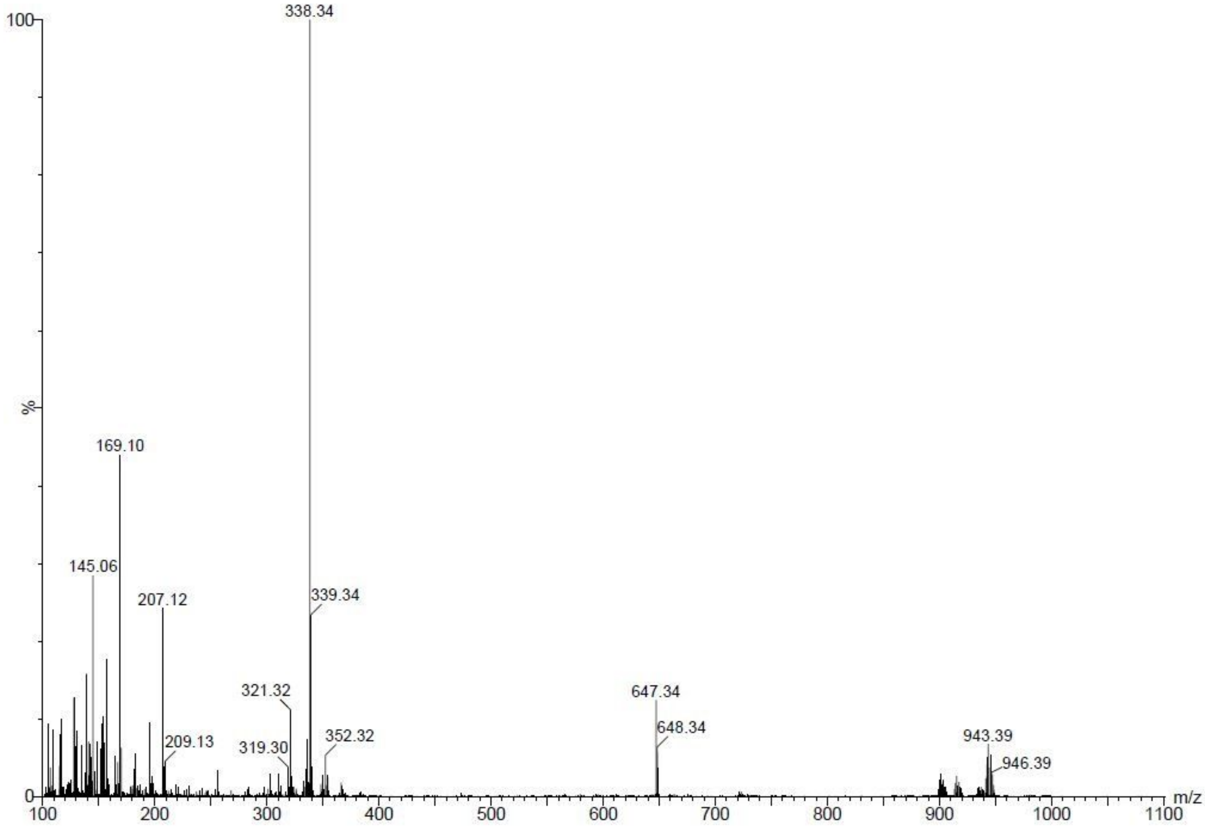

Figure S16. Mass spectrum of PdOEP4 before irradiation

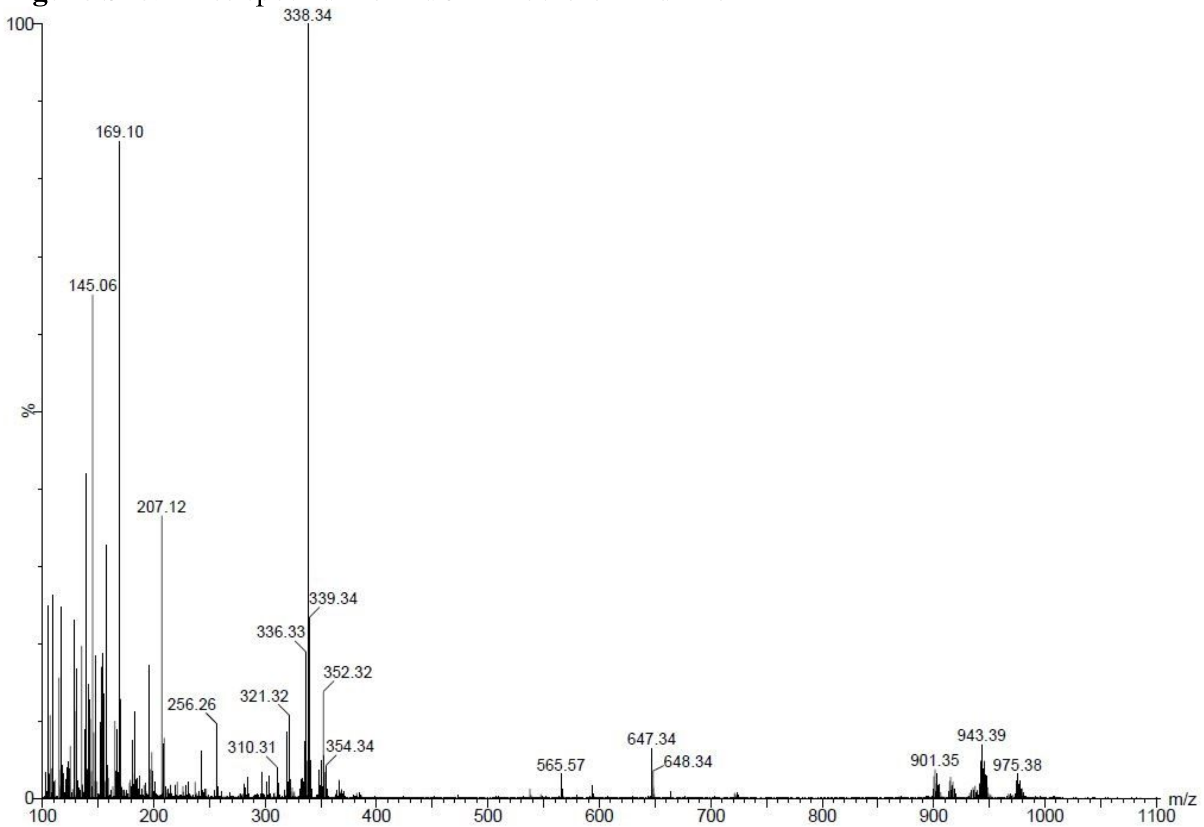

Figure S17. Mass spectrum of PdOEP4 after irradiation

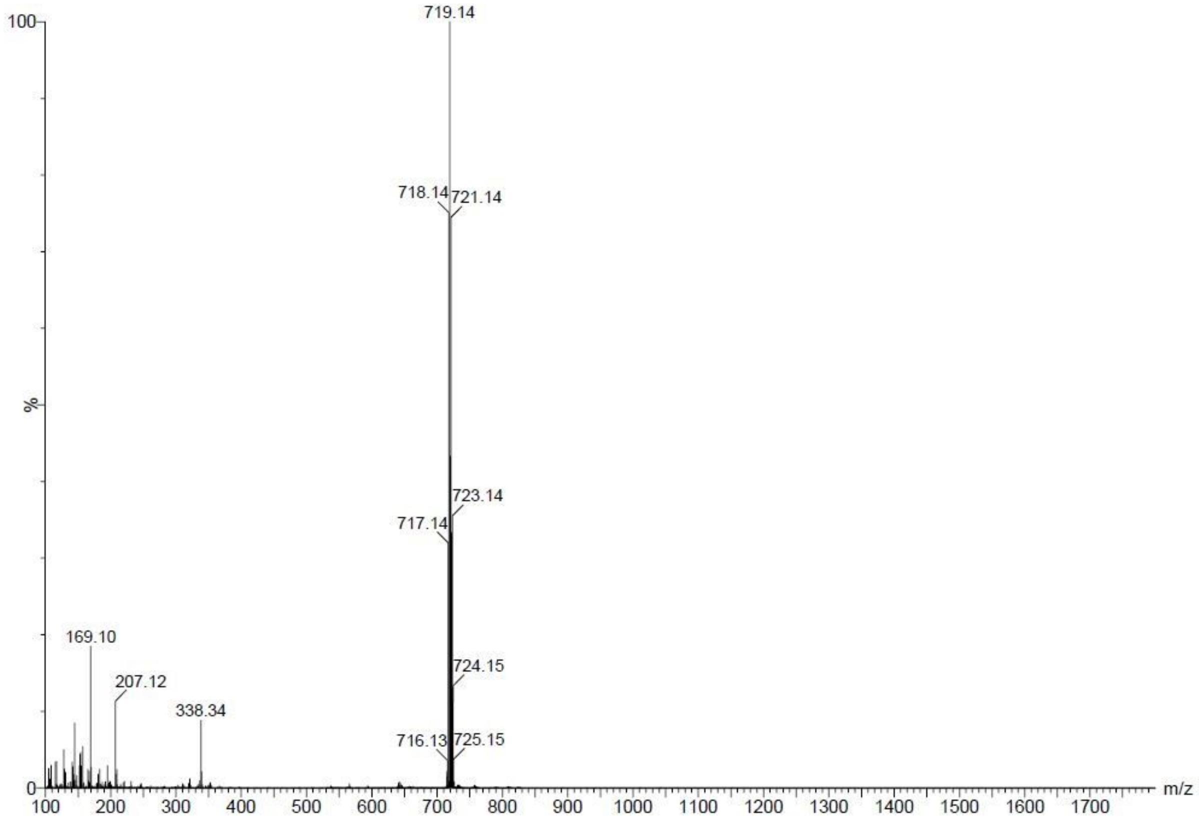

Figure S18. Mass spectrum of PdTPP before irradiation

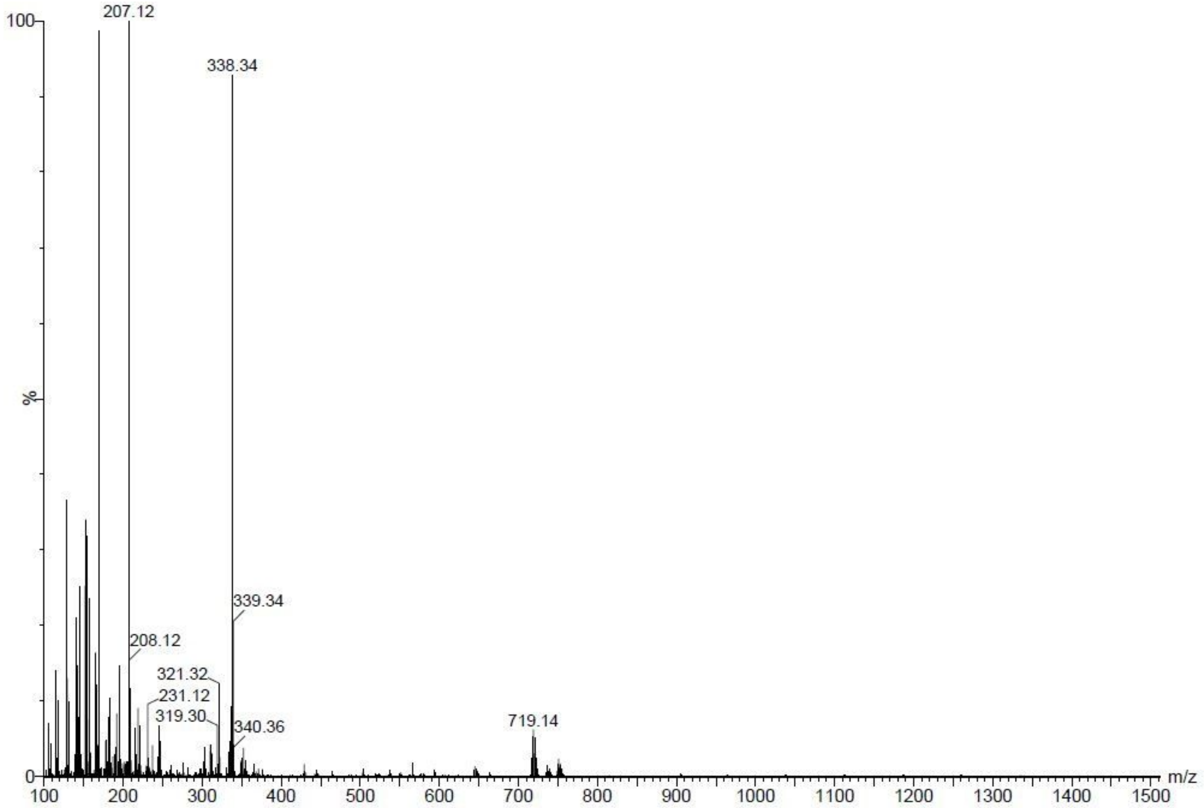

Figure S19. Mass spectrum of PdTPP after irradiation

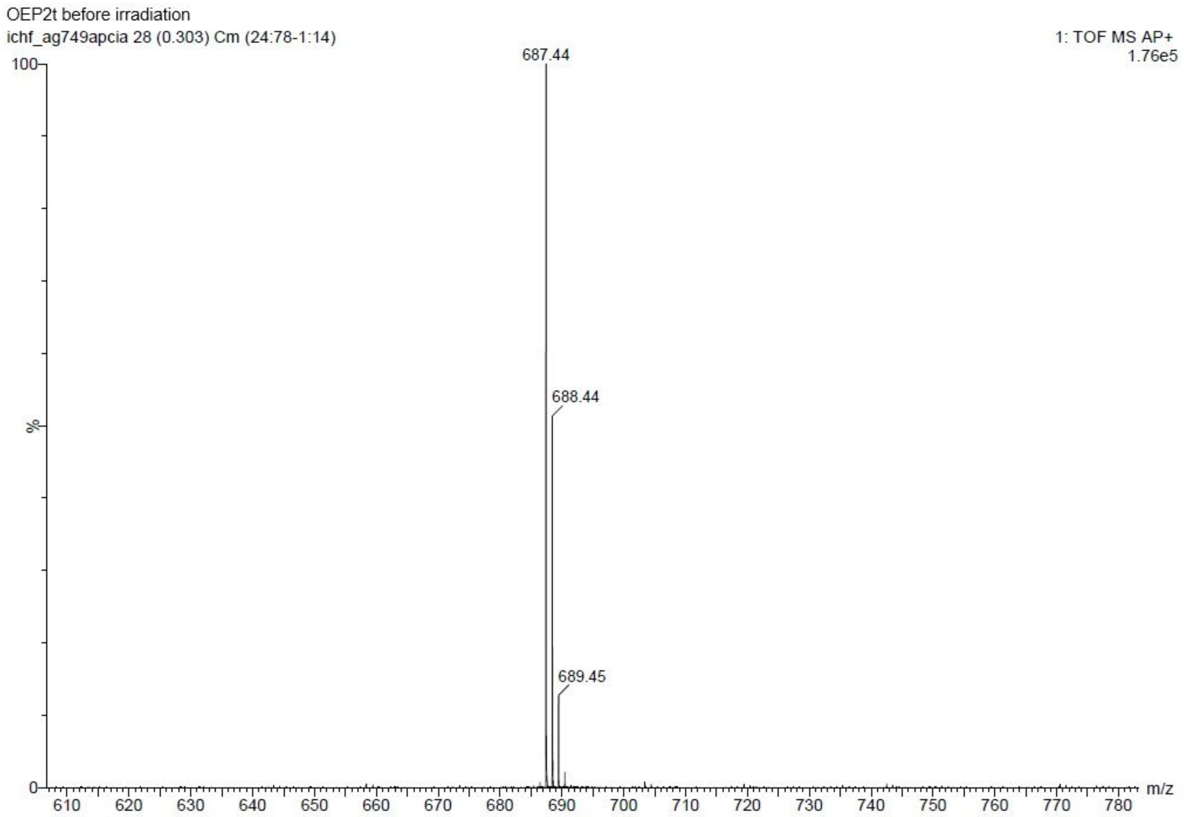

Figure S20. Mass spectrum of OEP2t before irradiation

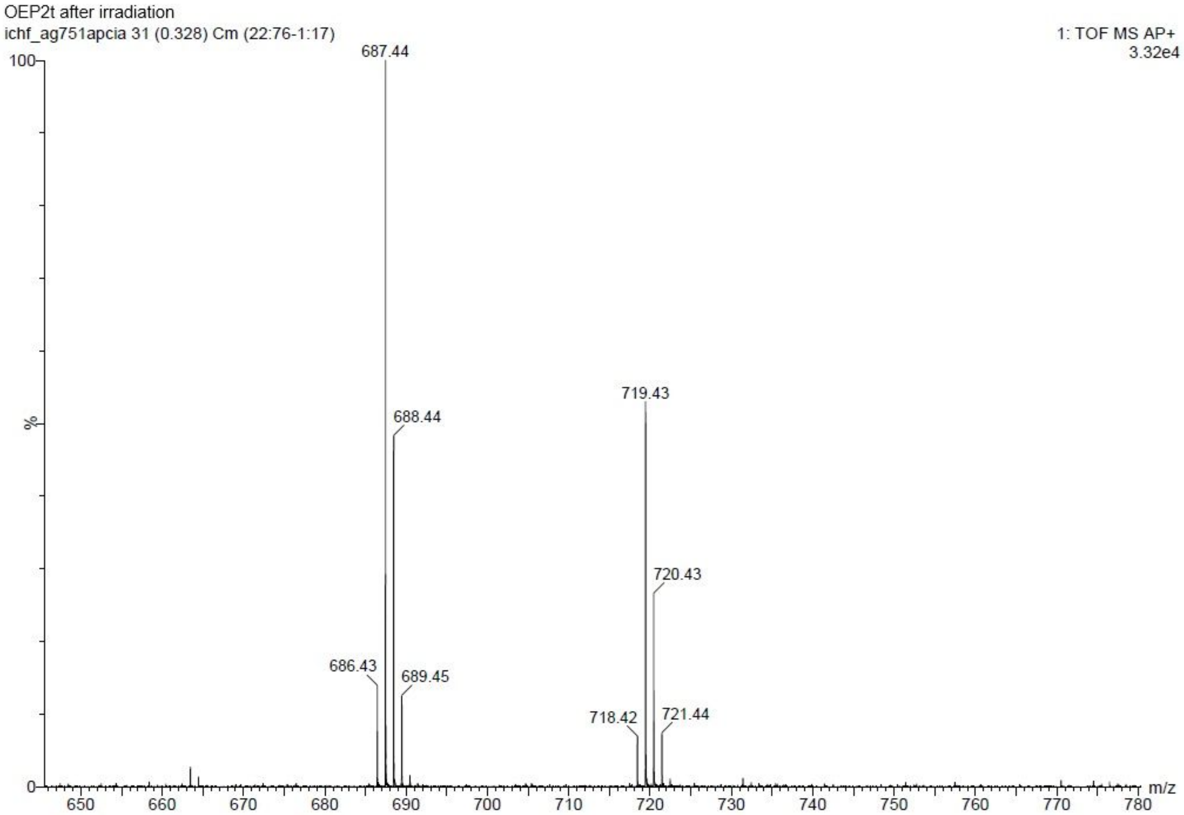

Figure S21. Mass spectrum of OEP2t after irradiation

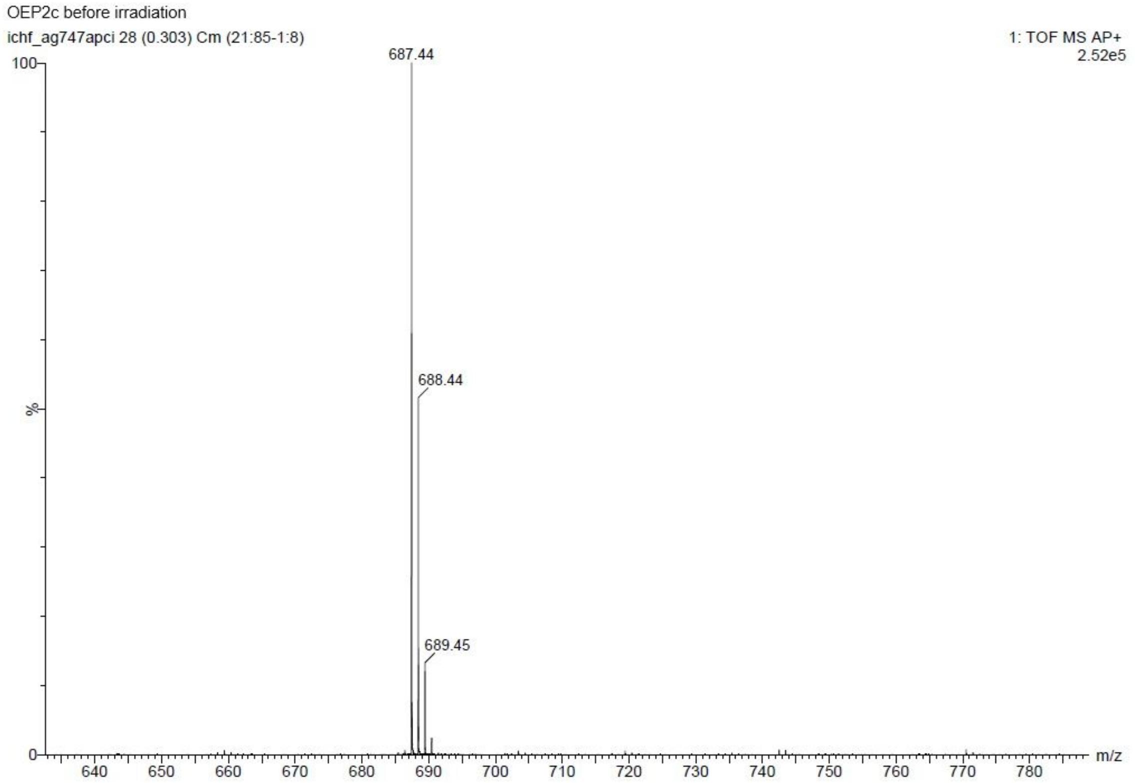

Figure S22. Mass spectrum of OEP2c before irradiation

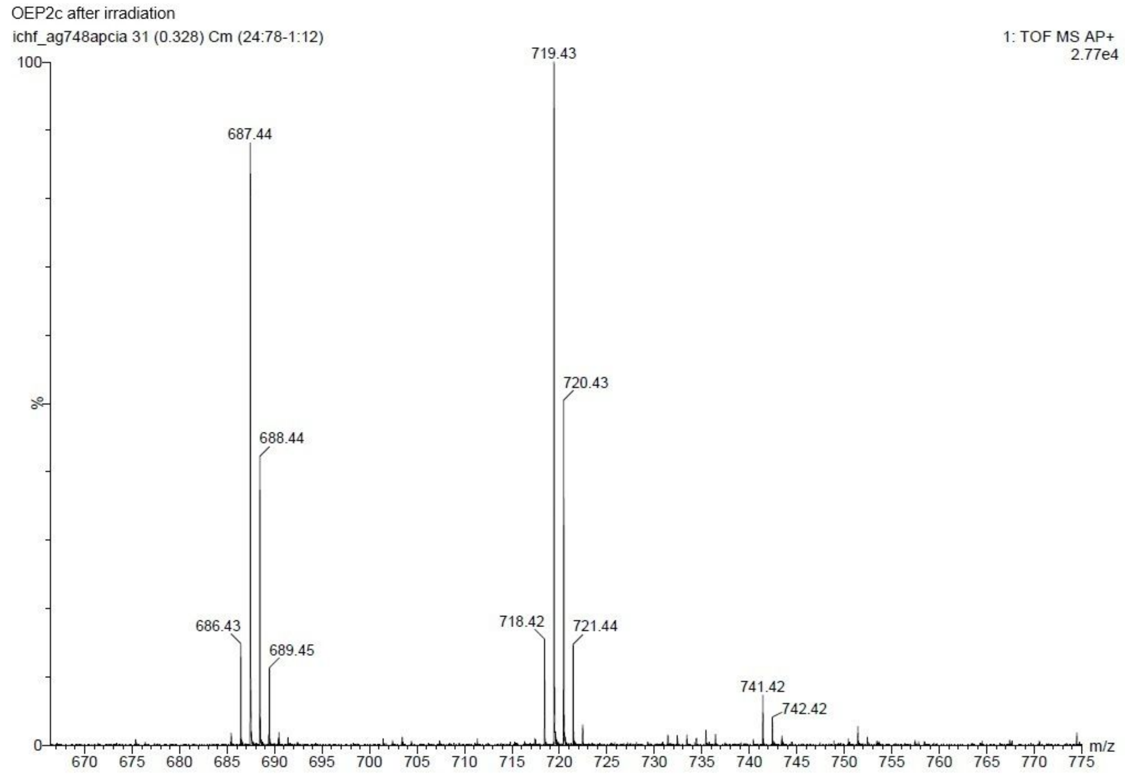

Figure S23. Mass spectrum of OEP2c after irradiation

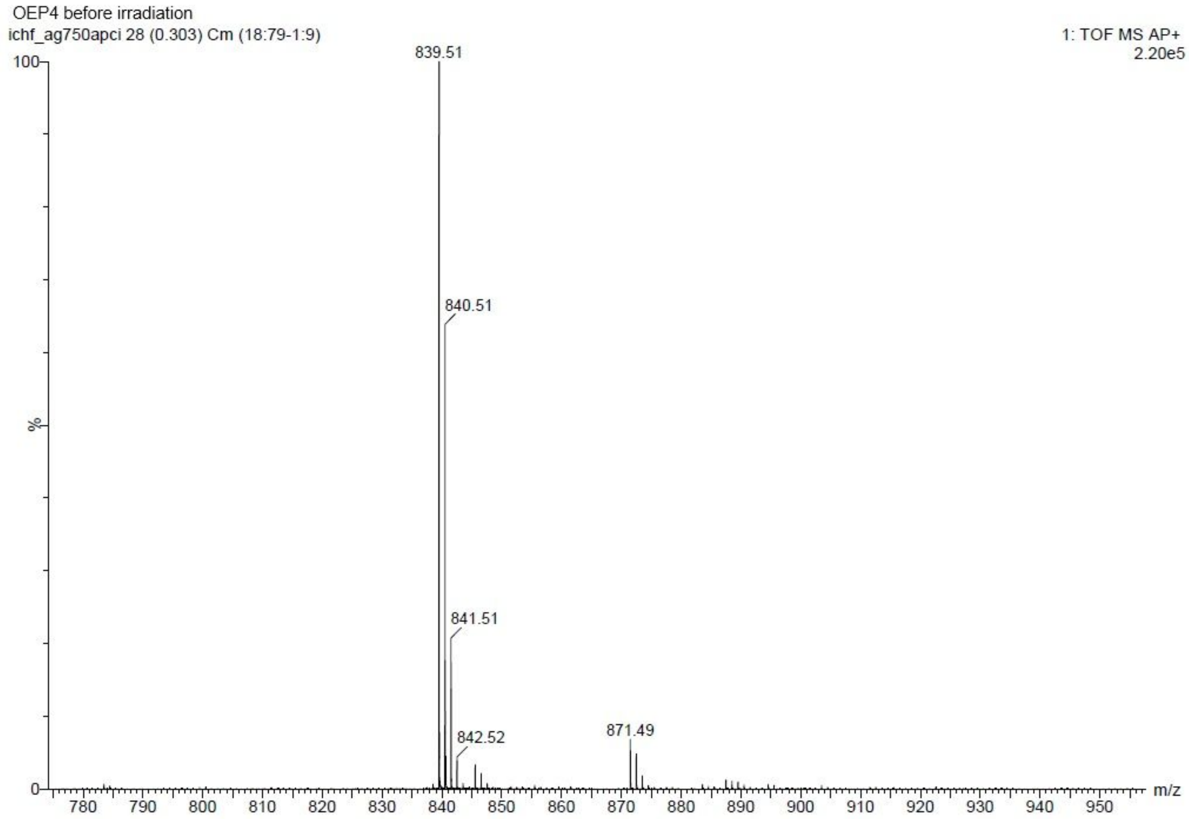

Figure S24. Mass spectrum of OEP4 before irradiation

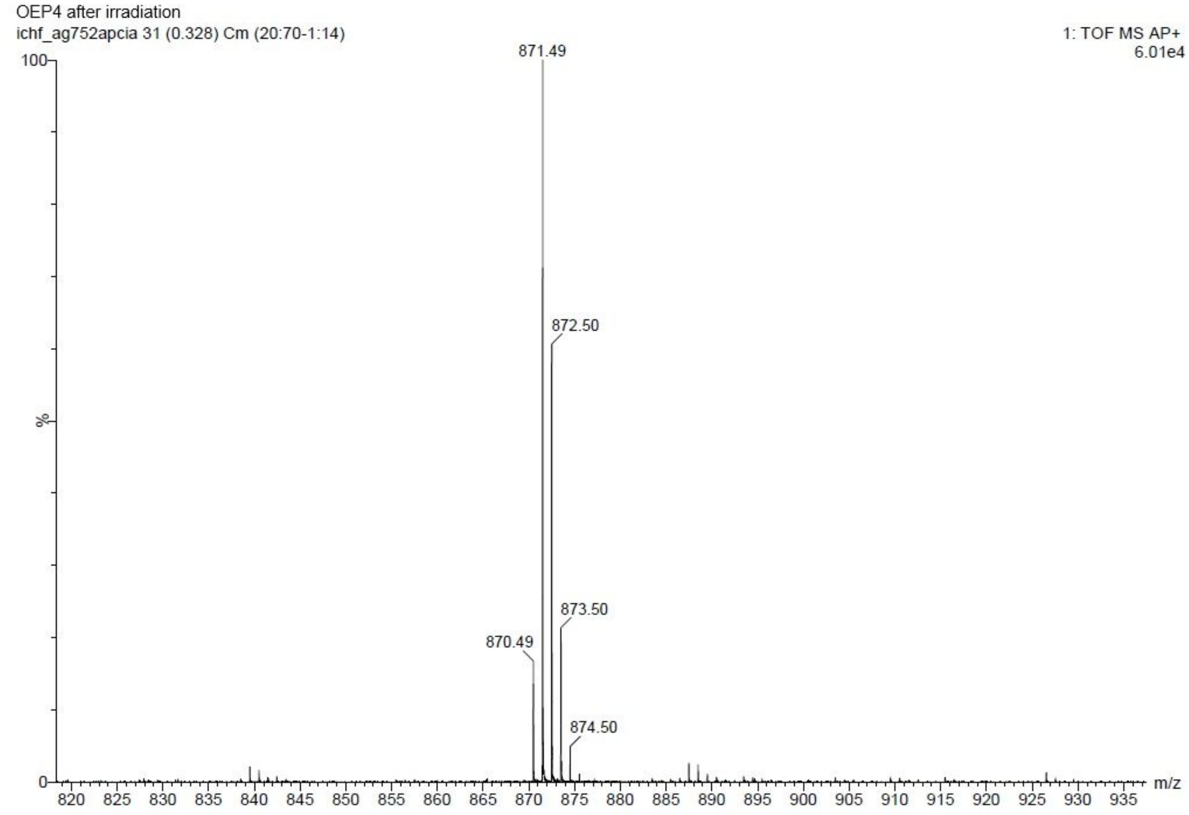

Figure S25. Mass spectrum of OEP4 after irradiation

Electrochemistry

PdOEP

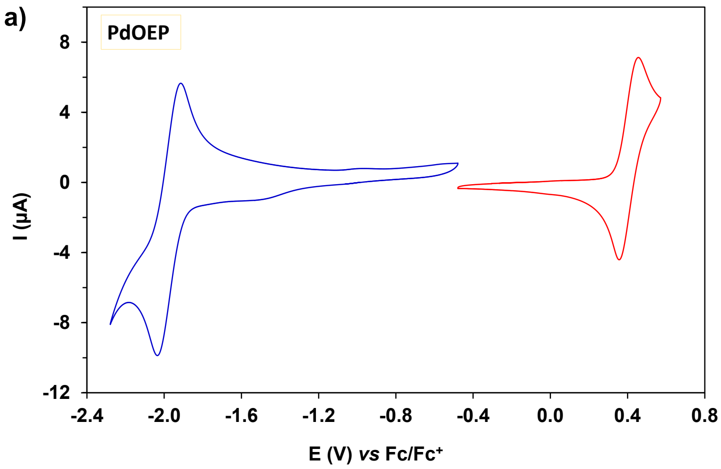

PdOEP1

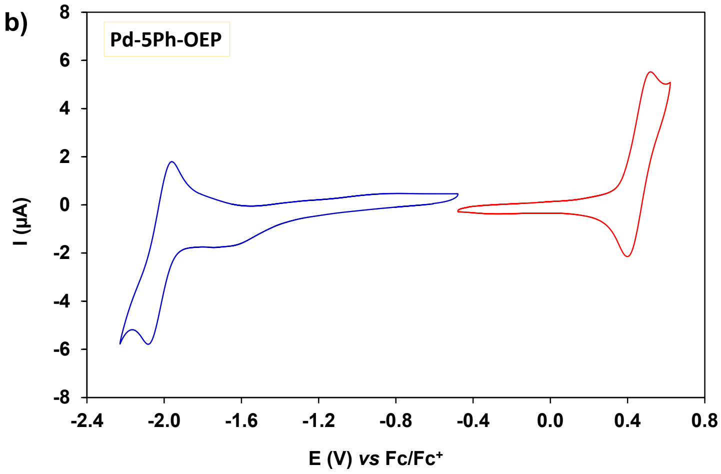

PdTPP

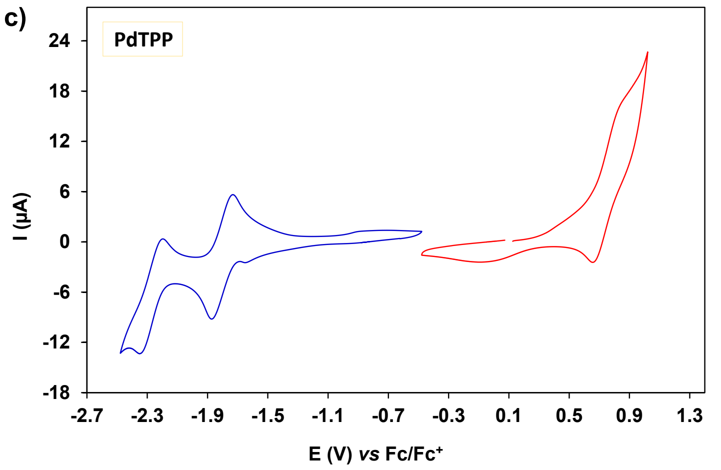

PdOEP4

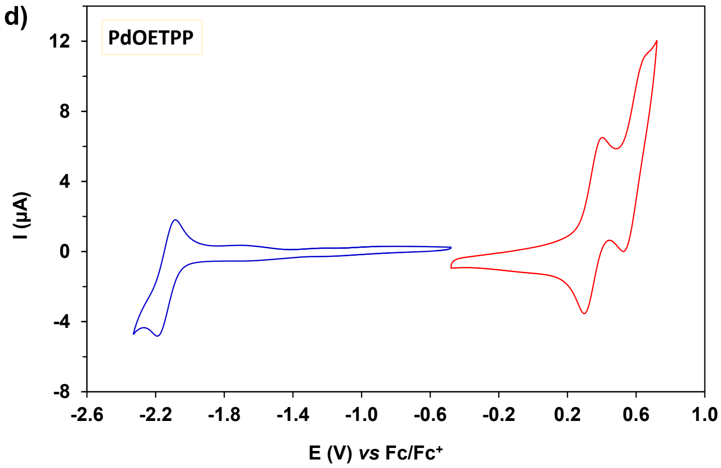

PdOEP2c

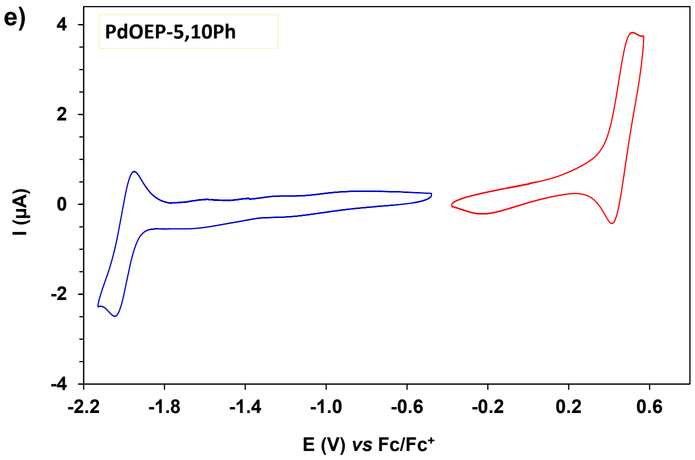

PdOEP2t

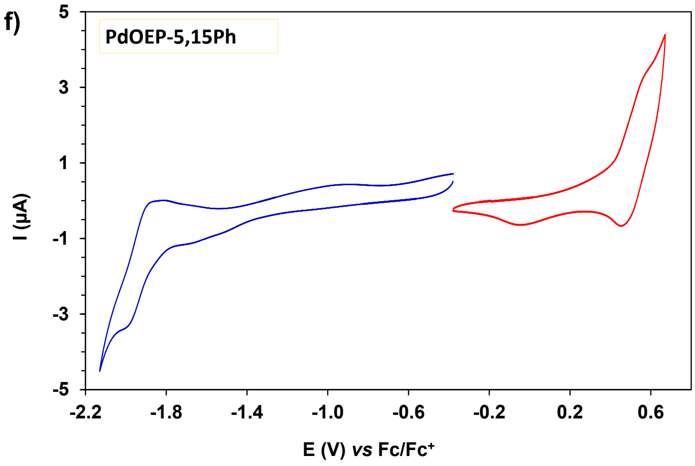

PdOEP3

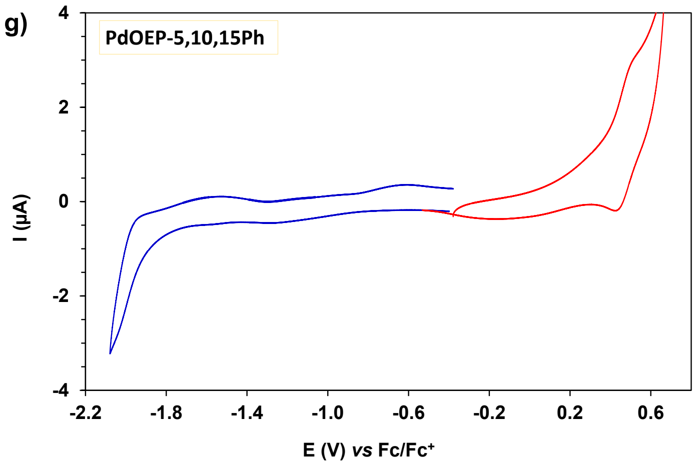

Figure S26. Cyclic voltammograms recorded for 1×10^{-3} M of PdOEP (a), PdOEP1 (b), PdTPP (c) and PdOEP4 (d), PdOEP2c* (e), PdOEP2t* (f), PdOEP3* (g). Electrolyte: 0.1 M Bu_4NPF_6 solution in THF; scan rate: 100 mV/s.

*The concentration of these compounds in the electrolyte solution is unknown.

Table S2. Cyclic voltammetry data of PdOEP, PdOEP1, PdOEP2t, PdOEP2c, PdOEP3 PdOEP4, and PdTPP. Potentials are given *vs* Fc/Fc$^+$ in [V].

Compound	Negative potentials range				E_{red1} (onset)	Positive potentials range				E_{ox3} (onset)
	E_{red1}	E_{ox1}	E_{red2}	E_{ox2}		E_{ox3}	E_{red3}	E_{ox4}	E_{red4}	
PdOEP	-2.04	-1.91	-	-	-1.90	0.46	0.36	-	-	0.34
PdOEP1	-2.08	-1.96	-	-	-1.94	0.51	0.4	-	-	0.36
PdOEP2t	-2.13	-	-	-	-1.85	0.67	0.46	-	-	0.38
PdOEP2c	-2.04	-1.95	-	-	-1.93	0.51	0.41	-	-	0.37
PdOEP3	-	-	-	-	-1.90	0.53	0.43	-	-	0.36
PdOEP4	-2.19	-2.08	-	-	-2.06	0.4	0.3	0.52	0.66	0.26
PdTPP	-1.87	-1.73	-2.35	-2.2	-1.71	0.86	0.66	-	-	0.61

Table S3. Ionization potentials (IP), electron affinities (EA) and energy gaps (Eg) of the studied compounds PdOEP, PdOEP1, PdTPP, PdOEP4, PdOEP12c, PdOEP2t, and PdOEP3. Data derived from cyclic voltammetry results and given in [eV].

Compound	IP	EA	E_g
PdOEP	5.18	2.59	2.59
PdOEP1	5.20	2.54	2.66
PdOEP2t	5.23	2.65	2.58
PdOEP2c	5.22	2.55	2.67
PdOEP3	5.20	2.59	2.24
PdOEP4	5.09	2.40	2.69
PdTPP	5.49	2.81	2.68

From the electrochemical data collected in **Tables 1** and **2** it is possible to estimate the ionization potential (IP) and the electron affinity (EA) of the molecules studied. In these cases potentials of the onsets of the first oxidation peak and the first reduction peak were used, following the equations recommended in [1]:

$$IP\ [eV] = |e|[1.15 \times E_{ox\ onset} + 4.79]\ [eV],$$

$$EA\ [eV] = e[1.18 \times E_{red\ onset} + 4.83]\ [eV].$$

As a result, the electrochemical band gap, E_g, was calculated as IP-EA.

Quantum-chemical calculations

Table S4. Calculated energies (in eV) of the highest occupied and lowest unoccupied MOs in S_0.

	HOMO-1	HOMO	LUMO	LUMO+1
PdOEP	-5.2937	-5.1057	-2.0871	-2.0871
PdOEP1	-5.2396	-5.0940	-2.0899	-2.0806
PdOEP2t	-5.1849	-5.0788	-2.0947	-2.0765
PdOEP2c	-5.1724	-5.0684	-2.1122	-2.0901
PdOEP3	-5.1111	-5.0521	-2.1195	-2.1089
PdOEP4	-5.0456	-5.0382	-2.1396	-2.1396
PdTPP	-5.4513	-5.3351	-2.3508	-2.3508
OEP2t	-5.1209	-5.0393	-2.1960	-2.1669
OEP2c	-5.0848	-4.9424	-2.2120	-2.1746
OEP4	-5.0537	-4.7117	-2.2466	-2.2259

Table S5. Calculated energies of the highest occupied and lowest unoccupied MOs in S_1.

	HOMO-1	HOMO	LUMO	LUMO+1
PdOEP	-5.2831	-5.1117	-2.1318	-2.0855
PdOEP1	-5.2222	-5.0989	-2.1152	-2.1067
PdOEP2t	-5.1650	-5.0845	-2.1119	-2.1108
PdOEP2c	-5.1463	-5.0755	-2.1369	-2.1367
PdOEP3	-5.0810	-5.0616	-2.1565	-2.1519
PdOEP4	-5.0513	-5.0113	-2.1889	-2.1837
PdTPP	-5.4853	-5.2722	-2.4175	-2.3739
OEP2t	-5.1223	-4.9963	-2.2387	-2.1680
OEP2c	-5.0902	-4.8714	-2.2575	-2.2158
OEP4	-5.1027	-4.6151	-2.4305	-2.2507

Table S6. Calculated energies of the frontier MOs in T_1.

	HOMO-1	HOMO	LUMO	LUMO+1
PdOEP	-5.3008	-5.0807	-2.1930	-1.9900
PdOEP1	-5.1863	-5.1457	-2.1720	-2.1089
PdOEP2t	-5.1348	-5.1215	-2.1786	-2.1065
PdOEP2c	-5.1416	-5.1035	-2.3095	-2.0286
PdOEP3	-5.1125	-5.0344	-2.2346	-2.1299
PdOEP4	-5.1093	-4.9672	-2.3911	-2.0335
PdTPP	-5.5351	-5.2450	-2.5549	-2.2896
OEP2t	-5.2310	-4.8905	-2.4354	-2.0278
OEP2c	-5.3406	-4.4864	-2.8447	-1.8743
OEP4	-5.2826	-4.3852	-2.7963	-1.9976

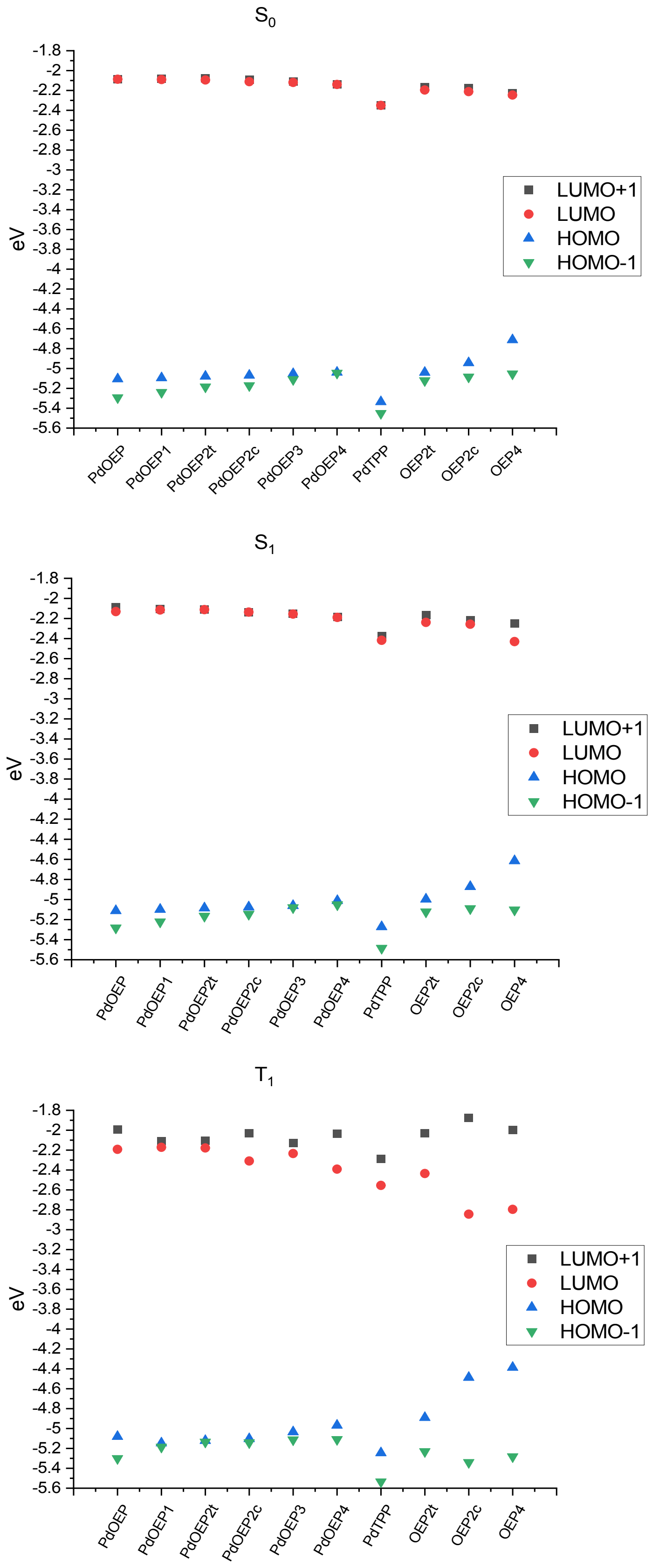

Figure S27. Calculated (B3LYP/def2SVP) energies of the frontier MOs in in S_0, S_1, and T_1 states.

Table S7. Dihedral angle C_α–C_m–C_α–N representing the degree of non-planarity in the optimized geometry of T_1 state (TD-DFT, B3LYP/def2SVP) of OEP2t, OEP2c, OEP4, and their Pd metallocomplexes.

	Dihedral angle, α (C_α–C_m–C_α–N) [deg]	Bond length, d (C_α–C_m) [Å]
OEP2t	10	1.458
OEP2c	58	1.488
OEP4	45	1.473
PdOEP2t	9	1.437
PdOEP2c	18	1.444
PdOEP4	25	1.448

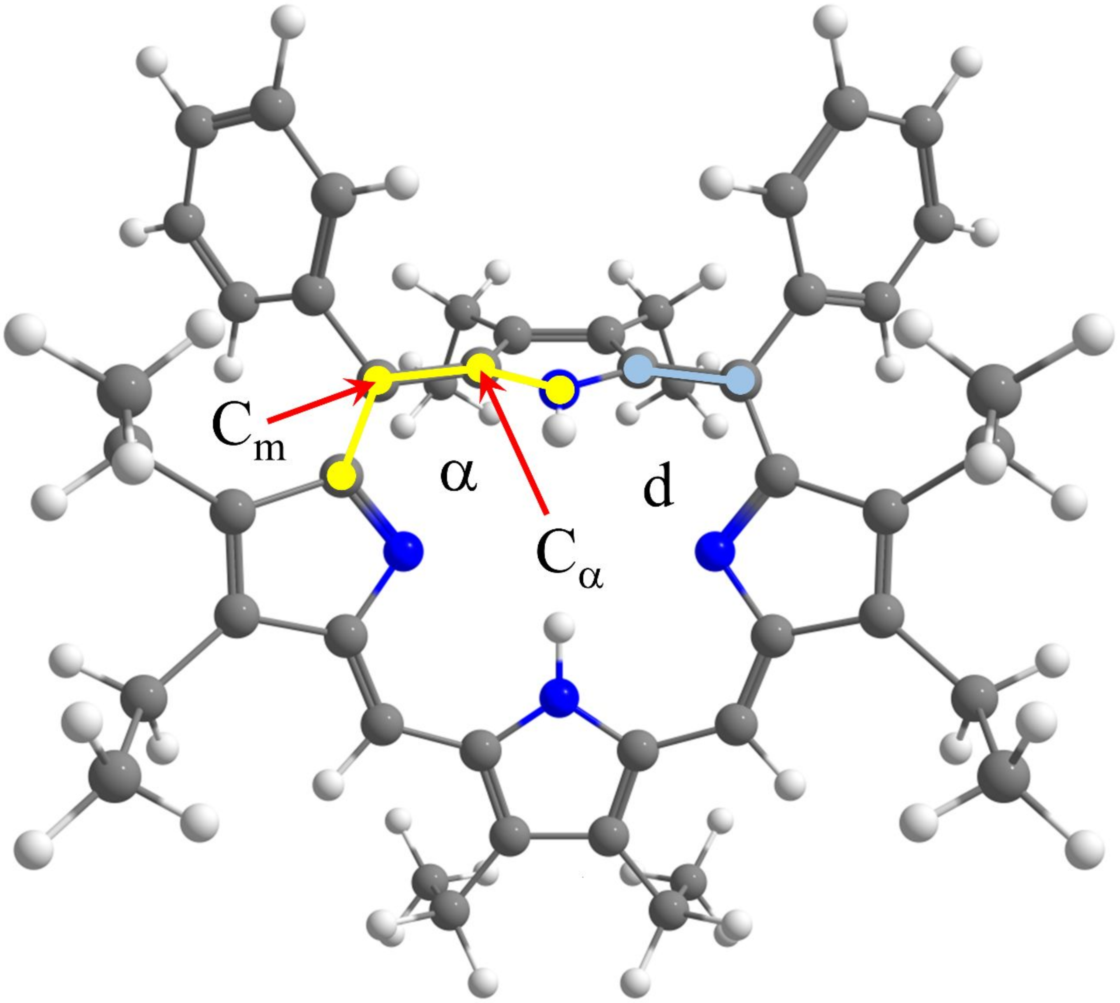

Figure S28. Dihedral angle α (C_α–C_m–C_α–N, atoms marked by yellow circles) representing the degree of non-planarity of the T_1 state of OEP2c and C_α–C_m bond, d (atoms marked by cyan circles).

Table S8. Calculated S_1 and T_1 states energies for optimized TD-DFT geometries and the experimental data.

	S_1, eV (nm)	T_1, eV (nm)	T_1^a, eV (nm)
PdOEP	2.45 (505)	1.84 (675)	1.89 (657/663)
PdOEP1	2.42 (512)	1.80 (689)	1.88 (660/668)
PdOEP2t	2.39 (519)	1.75 (708)	-
PdOEP2c	2.36 (525)	1.71 (726)	1.85 (671)
PdOEP3	2.31 (536)	1.67 (741)	1.80 (688)
PdOEP4	2.25 (551)	1.55 (799)	1.76 (705)
PdTPP	2.39 (520)	1.63 (762)	1.80 (689/669)

[a] – measured at 77K in methylcyclohexane-toluene mixture 5:1 / 295 K in toluene

Photophysical characteristics

Table S9. Quantum yields of photodegradation, Φ_{pb}[a], and Photobleaching rates, k_{pb}[b], determined for a series of PdOEP and OEP derivatives, PdTPP and TPP obtained in non-degassed and deaerated toluene.

Compound	Non-degassed toluene		Deaerated toluene		Non-degassed toluene				Deaerated toluene				
	Φ_{pb}	k_{pb} [s^{-1}][b]	Φ_{pb}	k_{pb} [s^{-1}]	Φ_{Δ}	Φ_{fl}	τ_{fl}, ns	τ_T, ns	τ_T, ns	k_r (10^7, s^{-1})	k_{nr} (10^7, s^{-1})	k_{ISC} (10^7, s^{-1})	K_Q^{O2} (10^9, M^{-1} s^{-1})
PdOEP	1.5×10^{-7}	0.62	3.0×10^{-5}	0.11	0.93	3.1×10^{-4}	-	241	270 000				1.7
OEP	4.5×10^{-7}	1.8	4.4×10^{-6}	0.03	0.75[d]	0.09[d]	12.4[d]	325	230 000	0.73	1.3	6.0	1.2
PdOEP1	6.4×10^{-7}	5.0	2.7×10^{-7}	1.4	0.54	2.8×10^{-4}	-	128	198				1.1
PdOEP2t	6.3×10^{-7}	4.8	1.2×10^{-7}	0.61	0.55	-	-	130	197				1.0
OEP2t	4.3×10^{-6}	17	8.3×10^{-7}	0.08	0.70	0.026	9	360	14 700	0.29	3.0	7.8	1.1
PdOEP2c	0.8×10^{-7}	1.3	$< 10^{-9}$	< 0.01	0.19[b]	2.3×10^{-4}	-	60	75				1.3
OEP2c	2.5×10^{-5}	6250	7.2×10^{-7}	103	0.02[e]	0.006	3	200	350	0.20	32.5	0.6	0.9
PdOEP3	2.1×10^{-7}	4.4	$< 10^{-9}$	< 0.02	0.12[b]	1.9×10^{-4}	-	48	57				1.3
PdOEP4	2.3×10^{-7}	7.7	$< 10^{-9}$	< 0.03	0.05[b]	1.6×10^{-4}	-	30	33				1.2
OEP4	1.3×10^{-3}	9507	1.1×10^{-6}	2.3	0.43	0.004	1.2	318	1100	0.3	47.1	36.0	0.9
PdTPP	4.9×10^{-7}	1.4	2.4×10^{-5}	0.09	0.9	2.0×10^{-4}	-	347	254 000				1.2
TPP	2.6×10^{-7}	2.0	2.0×10^{-6}	0.01	0.68	0.09[d]	9.7[d]	196	286 000	0.93	2.37	7.01	2.0

[a]estimated error: ±30%; [b]$k_{pb} = \Phi_{pb}/(\Phi_T \times \tau_T)$; τ_T is the triplet lifetime and Φ_T is the triplet formation yield.
[c]The triplet formation yield was assumed to be close to unity for all palladium metallocomplexes, for metal-free compounds Φ_T was assumed to be equal to Φ_{Δ}. [d]ref [1]

(1) Knyukshto, V. N.; Zenkevich, E. I.; Sagun, E. I.; Shulga, A. M.; Bachilo, S. M. Unusual pathways of triplet state dynamic relaxation in aryl-substituted porphyrins and their chemical dimers at 295 K. *J Fluoresc* **2000**, *10* (1), 55-68. DOI: 10.1023/A:1009439730497.
